# A genome-guided strategy for climate resilience in American chestnut restoration populations

**DOI:** 10.1073/pnas.2403505121

**Published:** 2024-07-16

**Authors:** Alexander M. Sandercock, Jared W. Westbrook, Qian Zhang, Jason A. Holliday

**Affiliations:** ^a^Genetics, Bioinformatics, and Computational Biology, Virginia Tech, Blacksburg, VA 24060; ^b^The American Chestnut Foundation, Asheville, NC 28804; ^c^Department of Forest Resources and Environmental Conservation, Virginia Tech, Blacksburg, VA 24060

**Keywords:** landscape genomics, recovery, temperate forests, breeding plans, whole genome sequencing

## Abstract

American chestnut (*Castanea dentata*) is a deciduous tree species of eastern North America that was decimated by the introduction of the chestnut blight fungus (*Cryphonectria parasitica*) in the early 20th century. Although millions of American chestnuts survive as root collar sprouts, these trees rarely reproduce. Thus, the species is considered functionally extinct. American chestnuts with improved blight resistance have been developed through interspecific hybridization followed by conspecific backcrossing, and by genetic engineering. Incorporating adaptive genomic diversity into these backcross families and transgenic lines is important for restoring the species across broad climatic gradients. To develop sampling recommendations for ex situ conservation of wild adaptive genetic variation, we coupled whole-genome resequencing of 384 stump sprouts with genotype–environment association analyses and found that the species range can be subdivided into three seed zones characterized by relatively homogeneous adaptive allele frequencies. We estimated that 21 to 29 trees per seed zone will need to be conserved to capture most extant adaptive diversity. We also resequenced the genomes of 269 backcross trees to understand the extent to which the breeding program has already captured wild adaptive diversity, and to estimate optimal reintroduction sites for specific families on the basis of their adaptive portfolio and future climate projections. Taken together, these results inform the development of an ex situ germplasm conservation and breeding plan to target blight-resistant breeding populations to specific environments and provides a blueprint for developing restoration plans for other imperiled tree species.

## Significance

Many forest tree species are vulnerable to the extreme effects of climate change, and in the absence of intervention, to extinction. Because of chronic fungal blight infections, American chestnut trees in the eastern United States are unable to reproduce, migrate, or evolve in response to climate change. Here, we explore the genomic basis of climate adaptation in American chestnuts, offering valuable insights for their conservation. We provide recommendations for preserving the adaptive legacy of the species and identify optimal planting locations for blight resistant breeding populations based on the adaptive composition of their respective genomes. This research provides specific guidance for the restoration of American chestnut and serves as a blueprint for conserving imperiled tree species facing similar challenges.

American chestnut (*Castanea dentata*) is one of the tree species most vulnerable to climate change in the United States (1). A large deciduous tree native to the Appalachian Mountains of eastern North America, the American chestnut was decimated by a fungal blight (*Cryphonectria parasitica*) beginning in the early 20th century, which killed billions of trees. While approximately 400 million individuals remain throughout the range, these are almost exclusively root collar sprouts that rarely achieve more than a few years of growth before being reinfected with blight (2, 3). This repetitive cycle of growth and die-back, and the lack of widespread reproduction, makes American chestnut functionally extinct in the wild (2).

The American Chestnut Foundation’s (TACF) efforts to develop blight resistant American chestnut through introgressive hybridization with Chinese chestnut (*Castanea mollissima*) followed by backcrossing to American chestnut (4), and the American Chestnut Research and Restoration Project’s (ACRRP) insertion of an oxalate oxidase (OxO) transgene, may soon enable widespread restoration plantings (5–7). However, particularly for genetically modified lines, a lack of genetic diversity in general, and adaptive diversity in particular, necessitates an outcrossing strategy to both increase effective population size and target specific families to the climate of local planting sites. And while the backcross breeding program has incorporated standing variation via pollination from wild trees, the extent of adaptive variation segregating in these populations is unknown.

Adaptation in temperate forest trees to local temperature and precipitation regimes may entail among-population differences in the timing of growth and dormancy transitions, maximum winter cold hardiness, water-use efficiency, and drought tolerance or avoidance (8, 9). Like many temperate tree species, American chestnut has migrated from glacial refugia to occupy extant ranges spanning thousands of kilometers from southern to northern limits (10). Differentiation in locally adaptive traits related to temperature variation has been documented in common gardens (11–13), which suggest adaptive clines similar to other widespread tree species. However, because of the rarity of wild reproduction, it is exceptionally difficult or impossible to assemble chestnut common gardens that encompass locally dense and geographically widespread sampling of the historical range. Hence, identifying the genomic targets of climate-related selection is a key step in understanding local adaptation in this species, as well as for developing strategies for conserving the relevant alleles and haplotypes. One way to approach this objective is through the concept of seed zones, whereby a species range is divided into the smallest number of regions that effectively describe how climate and ecoregions are partitioned. Ideally, these zones also reflect reaction norms from reciprocal common gardens, although such data are usually only available for species of high economic value. Existing climate and ecological data suggest >10 different seed zones within the historical American chestnut range (14, 15), but these may or may not reflect how adaptive traits and genomic diversity are arrayed across the landscape for chestnut in particular. Similarly, these zones do not inform the sampling intensity required to capture standing adaptive diversity.

Here, we describe the genomic basis of local adaptation in American chestnuts and make sampling recommendations to advance germplasm conservation efforts. We leverage a whole-genome dataset of ≈21 million single-nucleotide polymorphisms (SNPs) developed by Sandercock et al. (10), and additional whole-genome resequencing data for 371 American chestnut hybrids and wild trees, to i) identify loci significantly associated with climate; ii) summarize how these loci covary across the landscape to define seed zones for germplasm conservation; iii) estimate how these zones will shift under climate change; iv) develop sampling recommendations to capture wild adaptive diversity; v) characterize the extent to which backcross breeding has incorporated wild adaptive variation; and vi) perform simulations for introgressing wild adaptive diversity into backcross breeding populations.

## 1. Methods

### 1.1. Dataset Preparation.

**Fig. 1.**
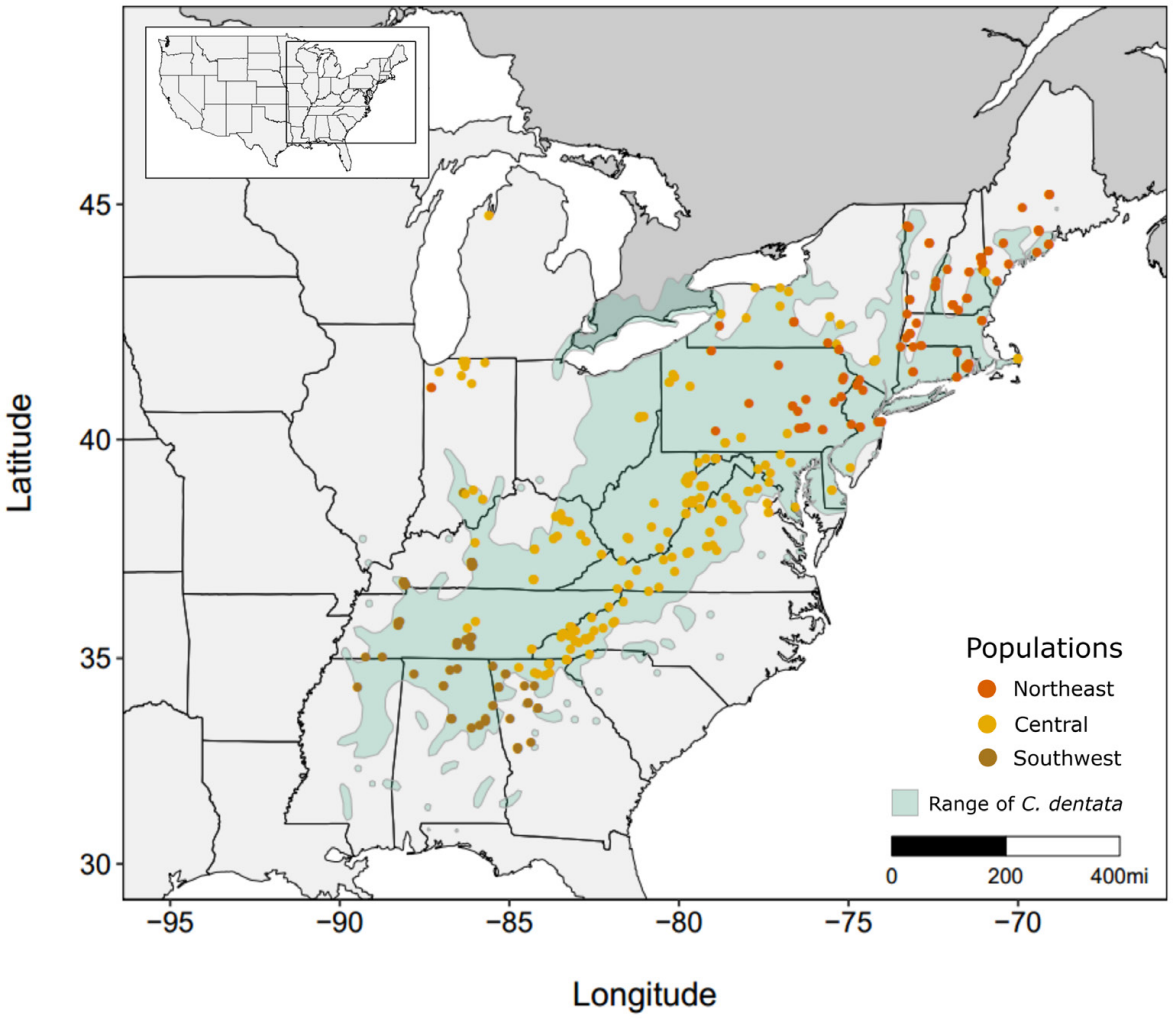
Range map of *C. dentata* and the locations of the 356 American chestnut samples and their assigned population from Sandercock et al. (10).

We used the 21,136,994 filtered SNPs and 356 *C. dentata* individuals that were previously reported by Sandercock et al. (10) (Fig. 1). Briefly, genomic libraries were prepared from 384 leaf samples collected throughout the natural range of American chestnut. These were sequenced at the HudsonAlpha Institute for Biotechnology on a NovaSeq 6000 in 150 bp paired-end mode and targeted a whole-genome coverage of 20X. After removing individuals with >10% missing data and >10% ancestry from other *Castanea* species, 356 *C. dentata* samples were retained (10). The final dataset had an average coverage of 17.3X. We removed sites with minor-allele frequency (MAF) less than 0.05 (16) and imputed missing data using BEAGLE v5.4 (17), leading to a final dataset composed of 11,526,713 SNPs across 356 samples.

Environmental data were obtained from climateNA (18) and comprised 32 monthly and seasonal climate variables (*SI Appendix*, Table S1). The environmental variables were the average for 1961 to 1990 and the ensemble projections under the CMIP6 future climate scenarios for 2080 (19, 20). Climate data were not available for three of the samples, and these were removed from the analysis. Collinearity between climate variables was assessed to exclude highly correlated variables that could increase false-positive rates in genotype–environment association analyses (21). To do this, we first used GF to rank the importance of each of the climate variables with a random subset of 50k SNPs (22, 23). Pearson’s correlations between each pair of climate variables were estimated using the pairs.panel() function from the *psych* R package (24). Climate variables were removed if they were highly correlated (R^2^ > 0.7) with another variable of higher importance from the GF predictor importance results.

We also performed whole genome sequencing of 269 backcross trees, 25 large-surviving (LSA) American chestnut trees, and 76 wild-type American chestnut trees conserved in TACF orchards. DNA was extracted using the Qiagen DNAeasy Plant DNA extraction kit and genomic libraries prepared using an Illumina DNA Prep Kit at the Duke University Center for Genomic and Computational Biology. Pools of 96 samples per lane were sequenced using an Illumina NovaSeq 6000 and four sequencing lanes. Bioinformatic analyses followed Sandercock et al. (10) and best practices of The Genome Analysis Toolkit (GATK) (25). Briefly, the fastq files were first aligned to the *C. dentata* reference genome using Burrows–Wheeler Aligner (BWA) mem with the -M and -R option (26) (*C. dentata* v1.1; http://phytozome-next.jgi.doe.gov/). Resulting SAM files were converted to sorted binary files (BAM) and indexed using SAMtools (27). PCR duplicates were marked with Picard (28) and candidate variants called with the GATK HaplotypeCaller algorithm, split by chromosome (29, 30). These GVCF files were combined using GatherVcfs into a single GVCF for each sample, which were then passed to GenotypeGVCFs to generate a raw variant call file (VCF), which was filtered for quality based on GATK best practices using VariantFiltration (25). Finally, BCFtools was used to remove INDELs, multiallelic SNPs, and sites with high missingness (>10% missing data) (27). The remaining missing sites were imputed using BEAGLE v5.4 (17).

### 1.2. Identifying Climate-Associated Adaptive Loci in American Chestnut Populations.

Redundancy analysis (RDA) and latent factor mixed models (LFMM) were used to test for relationships between SNPs and climate. RDA is a multivariate genotype–environment association (GEA) method that estimates the contributions of climate, geography, and population structure to patterns of genetic variation (31). For the RDA analysis, we used the vegan R package (32). First, we used a partial RDA (pRDA) to evaluate the contributions of climate, geography, and population structure to rangewide diversity. This analysis was computationally intractable with the full dataset, so we applied LD-pruning to the ≈11.5 million SNP dataset using PLINK v1.9 to remove SNPs with R^2^ values >0.5 within 50 SNP sliding windows (step size 10 SNPs), resulting in a reduced dataset of 2,934,239 SNPs (16). Using this dataset, we performed four tests: a full model with all factors, a climate-only model, a geography-only model, and a population structure–only model (21), and summarized the influence of each factor on genetic variation with an ANOVA in R. We then performed an RDA GEA scan of the ≈11.5M SNP dataset and the 10 uncorrelated climate variables following Capblancq and Forester (21). As correcting for population structure can cause the RDA outlier tests to be overly conservative, we report only the uncorrected model (31, 33). We retained three RDA axes, and the top 0.1% of *P*-values were identified as candidate locally adaptive sites.

As a complementary method to detect genotype–environment relationships, we used LFMM as implemented in the LFMM2 function of the R package LEA (34, 35). LFMM2 uses univariate regression to identify linear associations between loci and climatic variables, while accounting for confounding factors such as background allelic covariance arising from population structure (34). We first summarized the 10 uncorrelated climatic variables with PCA, retaining the first three PC axes, which explained ≈89% of variance within the climate variables dataset (*SI Appendix*, Table S2 and Fig. S1) (32). Like in the RDA GEA scan, we used the ≈11.5M SNP dataset for the LFMM GEA scan. The lfmm2() function in the LEA package in R was used to run LFMM2 three times (once for each environmental PC axis) with K = 2 latent factors to correct for population structure (35). *P*-values, z-scores, and the genomic-inflation factors (GIF) were estimated using the lfmm2.test() function with default parameters (35). To account for multiple testing, *P*-values were converted to q-values using the qvalue() function in R (36). Loci that had a q-value < 0.1 (FDR < 10%) were retained as candidate adaptive loci. Finally, outlier loci identified by the RDA and the three LFMM runs were combined for subsequent analyses.

To assess whether the putatively adaptive loci were enriched for coding or noncoding regions of the genome, the locations of outlier SNPs were categorized using SnpEff (37) and the American chestnut genome feature file (Cdentata_673_v1.1.gene_exons.gff3.gz; http://phytozome-next.jgi.doe.gov/). Introns were not included in the GFF file and were added using the agat_sp_add_introns.pl script from the AGAT toolkit (38). Promoters were also added to the GFF file as the 2 kb region upstream of the mRNA intervals.

### 1.3. Spatial Distribution of Adaptive Genomic Diversity and Genomic Offset.

To characterize the spatial distribution of adaptive genomic variation, we used gradientForest (GF) (23). GF estimates nonlinear turnover of allele frequencies (AFs) across environmental gradients. The shape of the nonlinear turnover functions estimated by GF enables rescaling of the environmental gradients to reflect the specific ways in which the multidimensional genomic variation is related to the climate space. We first estimated a GF model using the 18,483 adaptive SNPs from the LFMM and RDA analyses as response, and the climate information from the 10 uncorrelated climate variables for each sampling location as predictors. This trained model then predicted the spatial distribution of genomic variation over the entire chestnut range. A PCA was then performed on the GF model prediction and the top three principal components (PCs) were used to visualize the multidimensional adaptive genomic variation across the American chestnut range. To understand how climate change may impact the relationship between genome and environment, we performed the same procedure outlined above using two future climate predictions for 2080 (RCP4.5 and RCP8.5). We then calculated the Euclidean distance between the current climate GF model and the future climate model, which reflects “genomic offset”—an estimate of future maladaptiveness due to climate change.

### 1.4. Identifying Seed Zones Within the American Chestnut Natural Range and Their Change in Distribution Due to Climate Change.

A major objective of this research was to partition the chestnut range into geographic seed zones that reflect relatively homogeneous areas with respect to multivariate adaptive genomic variation, which will subsequently be used to conserve germplasm ex situ and guide breeding crosses that lead to climate-matched restoration populations. To do this, PCA loadings from the GF model above were clustered using the Optimal_Clusters_KMeans() function from the ClusterR package in R to determine the optimal number of seed zones (39, 40). Between K = 1 and K = 12 clusters were evaluated for fit to the data using AIC, sum of within-cluster-sum-of-squares-of-all-clusters (WCSSE), variance explained, and BIC. We then used the clara() function from the cluster R package with K = 3 (41).

To project genomic shifts between current and projected future climates, we first calculated Euclidean distances at 646,637 geographic locations (pixels) derived from a rasterized map of the chestnut range. For the 2080 climate change predictions, this range was extended to include potential habitat in the northeastern United States and southeastern Canada (*SI Appendix*, Fig. S2). As direct Euclidean pairwise comparisons across all pixels proved computationally intractable, we used the Fast Nearest Neighbor Search Algorithms and Applications (FNN) R package (42). Specifically, we used the knn.dist() function with k = 1 (number of nearest neighbors to search) to match each historical location (pixel) with its closest future counterpart based on the smallest genomic offset, effectively predicting the minimal migration path necessary to counteract the adverse effects of climate change. Centroids were separately calculated for the historical seed zone pixels and the uniquely matched future pixels for each seed zone. We then measured the migration distance between the historical and future seed zone centroids to estimate the average minimal migration distance for each seed zone.

### 1.5. Estimating the Number of Trees to Sample to Capture Wild Adaptive Diversity for Germplasm Conservation.

To estimate the number of trees required to sample from each seed zone to recapitulate the multivariate allele-frequencies (AFs) at adaptive loci and inform germplasm conservation, we used a custom Python script to compare AFs for adaptive SNPs in the full population with bootstrap samples of varying size using linear regression (https://github.com/alex-sandercock/Capturing_genomic_diversity). An R version of the Python script with parallel computation is available (https://github.com/alex-sandercock/castgen). R^2^ values from the regressions were used as a proxy for the percent diversity captured by each iteration, and samples were added until the R^2^ met or exceeded a target value (e.g., 90%). This process was repeated 100 times, and the number of trees to sample was averaged across all iterations. The result is an estimate of the mean number of trees to sample from each zone to meet the desired coefficient of determination between the adaptive multivariate AFs of the sample and that of the full dataset for each zone.

### 1.6. Local Adaptation Within the Breeding Population.

Since backcross American chestnut trees contain ancestry from throughout the natural range, predicting which seed zone they are most associated with based on their adaptive genomic content would help identify possible planting locations for backcross families, and provide information for which regions of the chestnut range are underrepresented in germplasm conservation orchards (GCOs). We performed a supervised ADMIXTURE analysis to infer the ancestry of each backcross sample attributable to the three seed zones and *C. mollissima* (43). To do this, we intersected the VCF files for the backcross breeding program, the wild American chestnut adaptive genotypes, and for 15 *C. mollissima* trees (10), which resulted in a dataset composed of 8,354 shared adaptive SNPs. The 356 wild American chestnut samples and the 15 *C. mollissima* were assigned seed zone and ancestry, and these were inferred for the backcross trees using ADMIXTURE. Backcross individuals with a majority assignment to a particular seed zone were then assigned to that seed zone.

To estimate the proportion of wild adaptive diversity represented by the breeding program, we intersected the backcross and wild sample datasets, which left 14,767 shared adaptive loci, and performed linear regressions of their AFs between the two groups using the matplotlib Python package (44). The resulting R^2^ provided an estimate of the percent of wild adaptive diversity captured by the breeding program. We also binned AFs for the wild and backcross samples to assess possible differences in the AF spectrum between these groups, which may not be apparent in the overall AF correlation.

Finally, we identified candidate regions for reintroduction of backcross trees based on their adaptive genomic composition. In the first two of three generations of backcrossing *C. dentata* × *C. mollissima* hybrids to *C. dentata*, the wild-type *C. dentata* parents came primarily from southwest Virginia. Additional adaptive diversity was incorporated in the third generation of backcrossing, where wild-type parents from all seed zones were used (4). To assess how mixed provenance ancestry of backcross trees potentially influences their climatic adaptation, we used Locator (45) to predict the locations where backcross trees had most similar AFs to the wild population for the ~14k sites that overlap between these datasets. We first estimated deviation in predicted location of the backcross trees from the location of their last wild parent. Then we compared the backcross v. wild parent deviations to the deviations in the Locator predictions of 356 wild trees’ locations in a cross-validation analysis. This comparison enabled the estimation of relative influence of mixed provenance ancestry versus methodological uncertainty in Locator location estimates.

### 1.7. Breeding Simulations between the Backcross and Wild American Chestnut Populations.

Breeding simulations were performed in AlphaSimR (46) to determine whether there would be gains in the percentage of adaptive alleles represented and correlations with wild-type AFs from breeding backcross trees with additional wild-type trees conserved in TACF’s GCOs. AlphaSimR requires haplotypes of the founding parents and genetic map positions of genotyped loci to simulate genotypes of progeny. Whole genome sequence data for 371 TACF backcross parents were subset to 322 parents with <50% missing genotypes and 18,483 climate-associated SNP loci. Genotypes were then imputed and phased in Beagle v5.4 (17, 47). The phased genotypes were split into two haplotypes per individual with the “cSplit” function from the “splitstackshape” R package. Genetic maps were generated from genotyping-by-sequencing data on 125 full sib progeny of two American chestnut parents (GMBig × Horn) (48). Two parental genetic maps were estimated in R/QTL (49) with the two-way pseudo-test cross method (50) after selecting markers with the expected segregation ratios and phase. The Horn parental map had the largest number markers (1,003 markers) and greatest coverage (99.5% of genome length) and was used to interpolate the genetic map positions for climate-associated markers using the program predictGMAP (https://github.com/szpiech/predictGMAP). Simulations were performed for each seed zone for the subset of sequenced backcross trees (Southwest N = 29, Central N = 165, Northeast N = 62) versus backcross + wild-type trees conserved in TACF’s GCOs (Southwest N = 52, Central N = 208, Northeast N = 62). A total of 30, 60, 90, or 120 randomly selected controlled crosses were made within each seed zone with the makeCross’ function in AlphaSimR to generate 10 progeny per cross. Simulations were repeated 10 times for each seed zone.

## 2. Results

### 2.1. Drivers of Genomic Variation Across the Range of American Chestnut.

We previously showed that the American chestnut range can be divided into three populations with longitudinal boundaries that roughly divide the species into southern, central, and northern groups (10). As these boundaries also track temperature and precipitation gradients, we predicted that population structure and climate would have the largest influence on genomic variation. The partial RDA models show that climate had the largest proportion of explainable variance (0.38), followed by population structure (0.13), and geography (0.08), and that all pRDA models were significant (*P* < 0.001, Table 1). The full pRDA model accounted for 11% of the total variance.

**Table 1.** Drivers of genomic variation in American chestnut

Partial RDA models	Inertia	Adj. R^2^	*P* (>F)	Proportion of explainable variance	Proportion of total variance
Full model	79,190	0.063	0.001***	1	0.11
Pure climate	30,117	0.012	0.001***	0.38	0.04
Pure structure	10,018	0.008	0.001***	0.13	0.01
Pure geography	6,368	0.003	0.001***	0.08	0.01
Confounded climate/structure/geography	32,687			0.41	0.05
Total unexplained	632,195				0.89
Total inertia	711,385				1

Four partial RDA models were performed to evaluate the influence that climate (10 environmental variables), population structure (first two PC axes from PCA on genomic dataset), and geography (geographic coordinates of each sample) had on the patterns of genomic variation throughout the American chestnut natural range. An ANOVA was performed after each partial RDA to estimate statistical significance. ***P ≤ 0.001.

**Fig. 2.**
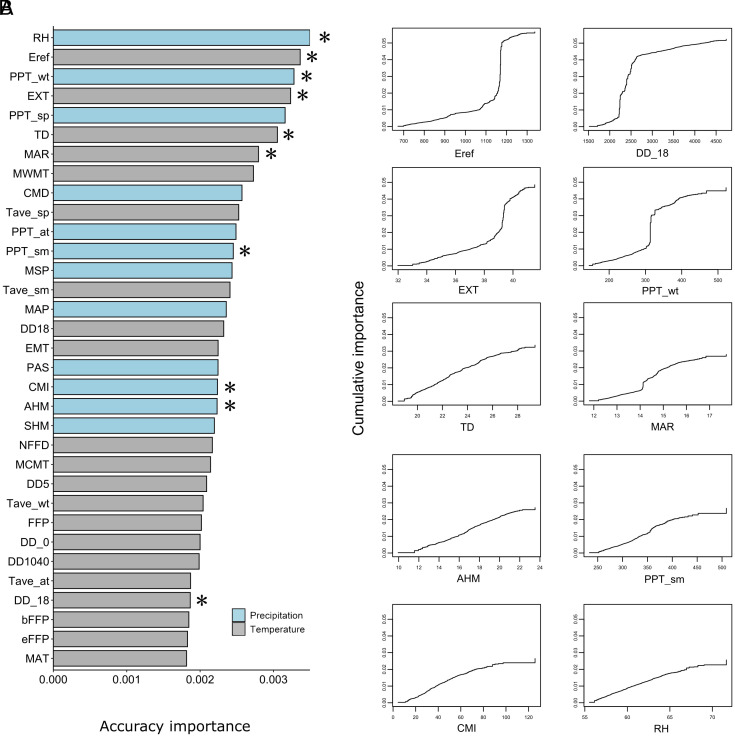
Importance of climatic variables in patterns of allelic turnover. (*A*) Ranked accuracy importance of 33 climateNA variables. Those denoted with “*” were used for the analyses. (*B*) Cumulative change in allele composition across the 10 climate gradients for 18,483 putatively adaptive loci.

We tested 10 uncorrelated climate variables for genotype–environment associations: **RH** [mean annual relative humidity (%)], **Eref** [Hargreaves reference evaporation (mm)], **PPT_wt** [winter precipitation (mm)], **EXT** [extreme maximum temp. over 30 y (°C)], **TD** [temp. difference between mean warmest month and mean coldest month (°C)], **MAR** [mean annual solar radiation (MJ m^−2^ d^−1^)], **PPT_sm** [summer precipitation (mm)], **CMI** [Hogg’s climate moisture index (mm)], **AHM** [annual heat-moisture index (mean annual temp. + 10)/(mean annual precip./1,000)], and **DD_18** (degree-days below 18 °C) (Fig. 2*A* and *SI Appendix*, Fig. S3). For LFMM, these 10 variables were summarized with PCA, and the three leading PCs were used as synthetic predictors. We identified 7,911 significant SNPs (FDR < 0.1) with LFMM: 4,988 associated with PC1 (MAR, PPT_wt, TD), 2,923 associated with PC2 (AHM, CMI, EXT), and no associations with PC3 (RH) (*SI Appendix*, Table S2). RDA analysis revealed 11,526 outlier SNPs (0.1% outliers), of which 954 were shared with LFMM (*SI Appendix*, Fig. S4), for a combined total of 18,483 putatively adaptive SNPs.

The genomic distribution of these SNPs was biased toward intergenic regions: 18.6% were located in promoter or genes, whereas in an AF-matched random dataset of the same size, 28.6% of SNPs were located in promoters or genes (*SI Appendix*, Table S3). Adaptive SNPs were nonrandomly distributed among chromosomes, with chromosomes 6, 10, and 12 having significantly more adaptive SNPs than expected under the null of even distribution relative to the number of tested SNPs per chromosome (P ~ 0 based on exact binomial test in R; *SI Appendix*, Table S4). Among genes, 433 contained at least one adaptive site, and chromosomes 6, 10, and 12 were similarly enriched for these “adaptive genes” (*SI Appendix*, Table S4).

Among climate variables, allelic turnover was relatively linear for temperature differentials (TD), annual heat:moisture indices (AHM), summer precipitation (PPT_sm), Hogg’s climate:moisture indices (CMI), and mean annual relative humidity (RH) (Fig. 2*B*). By contrast, sharp turnovers occurred at evaporative demand (Eref) between 1,150 and 1,200 mm, extreme temperatures (EXT) between 39 and 40 °C, cooling degree-days (DD_18) between 2,000 and 2,500 d, and winter precipitation (PPT_wt) between 300 and 350 mm (Fig. 2*B*). Finally, mean annual solar radiation (MAR), showed a slight increase in allele turnover rate between 14 and 15 (MJ m^−2^ d^−1^) (Fig. 2*B*).

### 2.2. Geographic Distribution of Genomic Variation and (Mal)Adaptation Under Current and Future Climates.

**Fig. 3.**
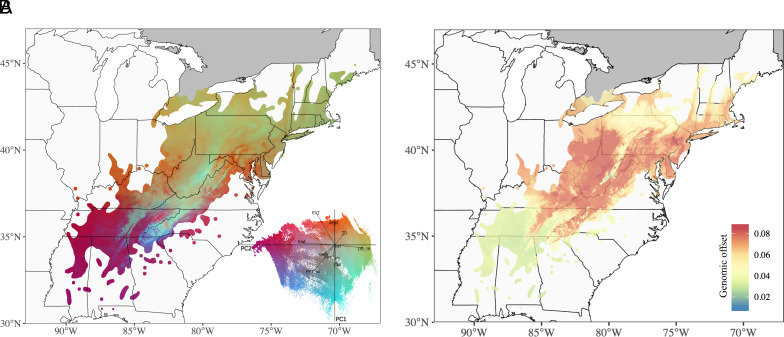
Adaptive genomic diversity under past and future climate conditions. (*A*) Spatial distribution of genomic variation across the American chestnut natural range. PC loadings from the GF model of 10 uncorrelated climate variables from 1961 to 1990 environmental data and 18,483 putatively adaptive loci. *Inset*: biplot of loadings for PC1 and PC2. (*B*) Genomic offset under 2080 climate projections for severe emissions (RCP8.5) scenarios.

Our genotype–environment analysis suggested differences in the climatic drivers of adaptive genomic variation between northern and southern portions of the species range. Northern portion of the range was associated with temperature and precipitation (TD, DD_18, AHM); southern portion of the range with evaporative demand and extreme temperatures (Eref and EXT); and high elevations in the Appalachian Mountains of North Carolina, Virginia, and West Virginia were associated with precipitation and solar radiation (CMI, MAR, PPT_sm, and PPT_wt) (Fig. 3*A*). To understand how climate change will impact the match between current and future climates, we estimated genomic offsets for 2080 for both middle and high emissions climate scenarios (RCP4.5 and RCP8.5, respectively). Under both scenarios, the central portion of the American chestnut range was predicted to experience elevated maladaptation, effects that were exaggerated for the high emissions scenario (Fig. 3*B* and *SI Appendix*, Fig. S5).

### 2.3. Defining Seed Zones and Predicting Climate-Driven Range Shifts.

**Fig. 4.**
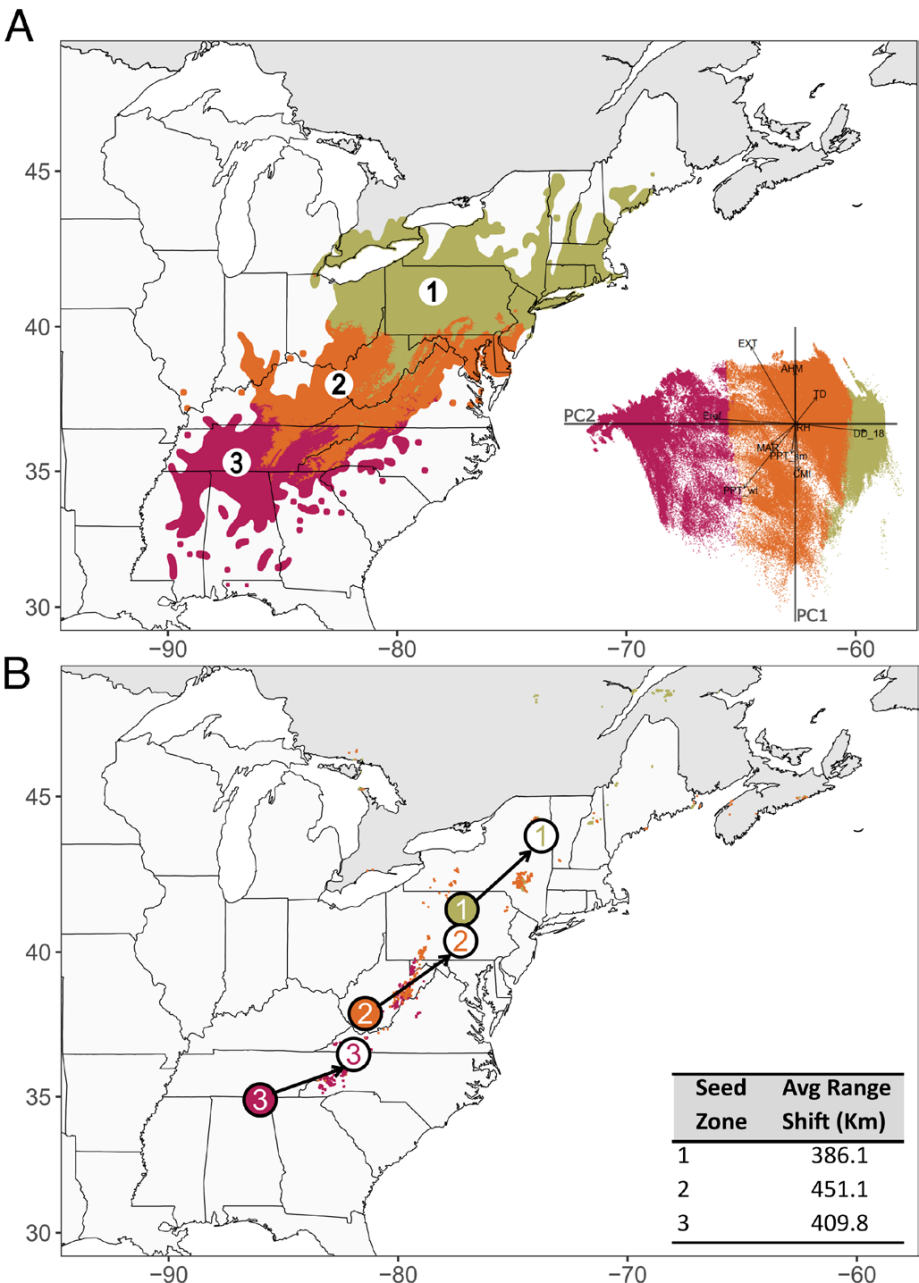
GF predicted seed zones for American chestnut. (*A*) Spatial distribution of the three zones across the historical species range. *Inset*: PCA biplot of GF-predicted genomic variation. (*B*) Projected range shifts for each seed zone to minimize genomic offset under climate change. Solid colored circles are centroids of the historical seed zones, while white background circles are centroids of projected shifts under 2080 RCP 8.5 conditions. The colored pixels are the projected migration locations for each seed zone where the genomic offset was lowest under the future climate predictions (red = Seed Zone 3, orange = Seed Zone 2, green = Seed Zone 1). *Inset*: The distance in kilometers between the historical and projected centroids for each seed zone.

As climate was the greatest driver of genomic variation, we hypothesized that we could partition the range into seed zones with boundaries reflecting precipitation and temperature gradients in the eastern US. Model selection with GF of between 1 and 12 climate-based seed zones suggested that two or three zones were most compatible with the data (*SI Appendix*, Figs. S6–S9). With three such zones, the range was split into northern, central, and southern regions, with latitudinal boundaries at approximately 36N and 39N (Fig. 4*A*). We hereafter refer to these northern, central, and southern seed zones as SZ.1, SZ.2, and SZ.3, respectively. Genetic differentiation (F_ST_) among these seed zones was approximately 5.5- to 6.5-fold higher at climate-associated loci compared with neutral loci (*SI Appendix*, Fig. S10).

To minimize the impacts of climate change, the three seed zones’ geographic centroids required shifts at least 300.4 km and 386.1 km northeast from their historical distribution for the moderate and high climate change scenarios, respectively (Fig. 4*B* and *SI Appendix*, Fig. S11). For the moderate scenario, the SZ.3 centroid was predicted to require the greatest movement (334.9 km), followed by SZ.1 (320.9 km), and SZ.2 (300.4 km) (*SI Appendix*, Fig. S11). For the severe scenario, the largest movement was required for SZ.2 (451.1 km), followed by SZ.3 (409.8 km) and SZ.1 (386.1 km) (Fig. 4*B*). Centroids for the three seed zones required shifts of 65.2 to 150.7 km farther for the severe scenario compared with the moderate scenario.

### 2.4. Sampling to Capture Wild Adaptive Variation.

Previous estimates of genomic diversity in American chestnut show that the southern portion of the range had the highest levels of heterozygosity, which decreased as latitude increased (10). As such, we expected that sampling intensity required to capture most adaptive diversity would be lower in the more southern SZ.3, and would increase with latitude. Consistent with this, we found that the fewest trees will need to be sampled from SZ.3, and the most from SZ.1, to match multivariate adaptive AFs for each zone at the 90% and 95% coefficient of determination thresholds (Table 2). An approximately 10-fold difference in sampling intensity will be required to match AFs at the 99% threshold compared with 90% (Table 2). Using a three seed zone model will require more trees to be sampled overall (90% = 38.15 trees, 95% = 76.75 trees, 99% = 370.35 trees) compared with the two seed zone model (90% = 27.78 trees, 95% = 56.03 trees, 99% = 274.93 trees) or the one seed zone model (90% = 26.86 trees, 95% = 55.89 trees, 99% = undetermined) (Table 2 and *SI Appendix*, Tables S5 and S6).

**Table 2.** Sampling estimates to capture adaptive diversity from each American chestnut wild seed zone population

Seed zone	% variance explained	# of trees to sample	95% CI
1	90	14.50	(13.35, 15.65)
2	90	13.42	(12.27, 14.57)
3	90	10.23	(9.41, 11.05)
1	95	28.30	(26.33, 30.27)
2	95	27.74	(25.63, 29.85)
3	95	20.71	(19.32, 22.10)
1	99	132.33	(124.61, 140.05)
2	99	136.34	(127.60, 145.08)
3	99	101.68	(94.76, 108.60)

### 2.5. Adaptive Diversity Captured Within the Backcross Breeding Program.

**Fig. 5.**
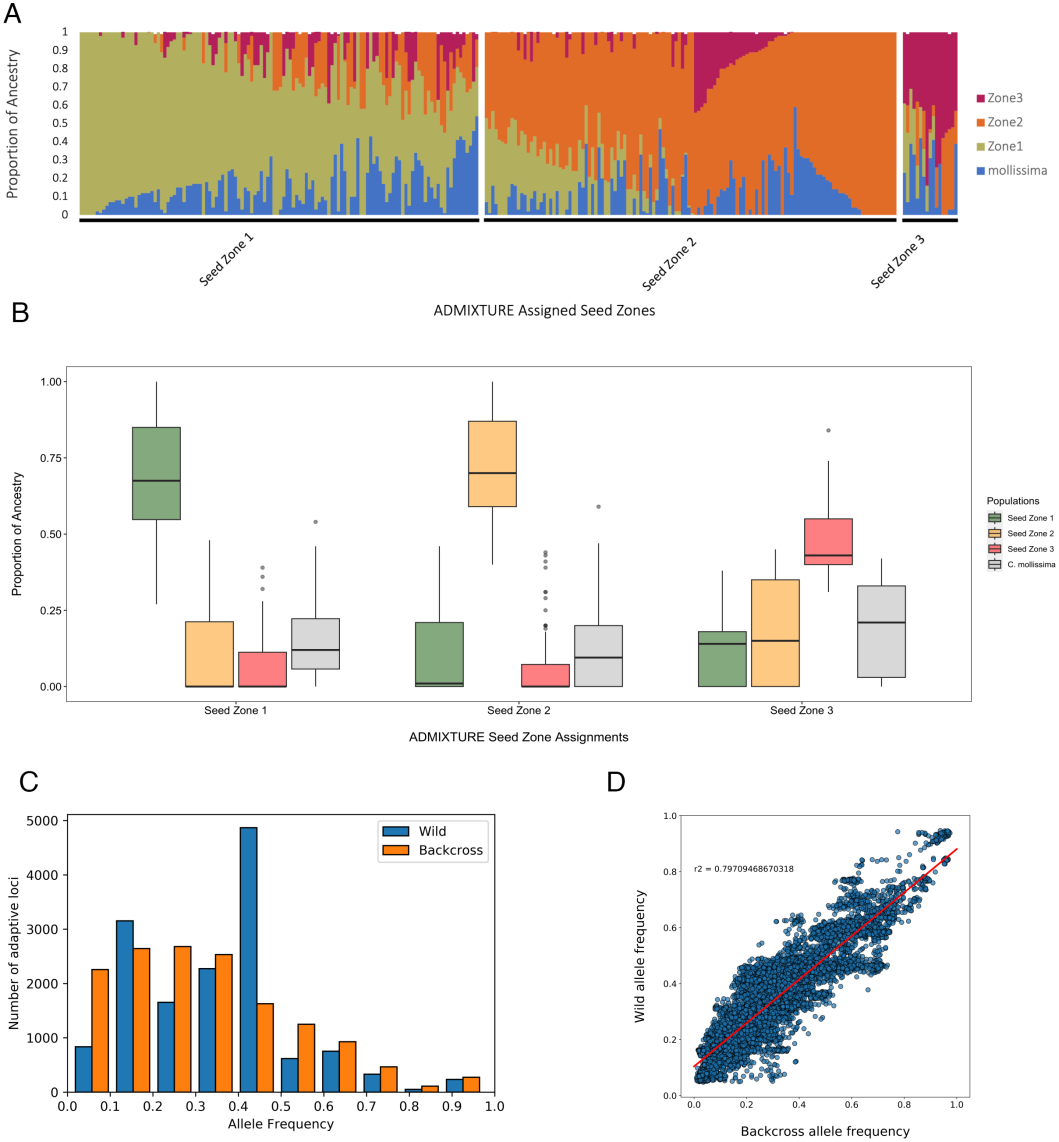
Wild adaptive diversity captured by the TACF backcross breeding program. (*A*) Supervised ADMIXTURE plot of ancestry. Each vertical line is a single backcross tree and the different colors represent the proportion of ancestry from each population associated with that tree. (*B*) Average proportion of ancestry within each assigned seed zone from backcross trees. (*C*) Allele-frequency spectrum at shared adaptive loci for the wild and backcross populations. (*D*) Linear regression of AFs at shared adaptive loci for the wild and backcross populations.

In the first two generations of backcrossing *C. dentata* × *C. mollissima* hybrids with *C. dentata*, the wild-type *C. dentata* fathers primarily came from southwestern VA, SZ.2. For crosses leading to the third backcross generation, TACF’s state chapters incorporated wild-type *C. dentata* parents from throughout the historical range. Based on this breeding design, we hypothesized that there would be a bias toward SZ.2 ancestry within the backcross samples. Indeed, ancestry for the core TACF and central state chapter breeding program material was predominantly from SZ.2, while SZ.3 was the least represented (mean SZ.1 ancestry = 37.4%, mean SZ.2 ancestry = 40.2%, mean SZ.3 ancestry = 8.8%, mean *C. mollissima* ancestry = 13.6%). Approximately 76% of the backcross trees contained ancestry from at least two seed zones (Fig. 5 *A* and *B*).

The allele-frequency distribution for adaptive loci within each seed zone was similar to their associated backcross trees, but the highest correlation was between wild and backcross trees of SZ.2, with ≈84% of the AF variation in the wild SZ.2 population explained by that of the backcross SZ.2 associated trees (*SI Appendix*, Fig. S12). The overall allele-frequency distribution for adaptive loci in wild and backcross samples was skewed toward medium frequency alleles in the wild population, and low frequency alleles in the backcross population (Fig. 5*C*). Nevertheless, the overall AF distribution correspondence was high between wild and backcross populations: ≈79.8% of AF variation in the wild population can be explained by that of the backcross population (Fig. 5*D*).

### 2.6. Matching Adaptive Niche of Backcross Trees for Restoration Plantings.

**Fig. 6.**
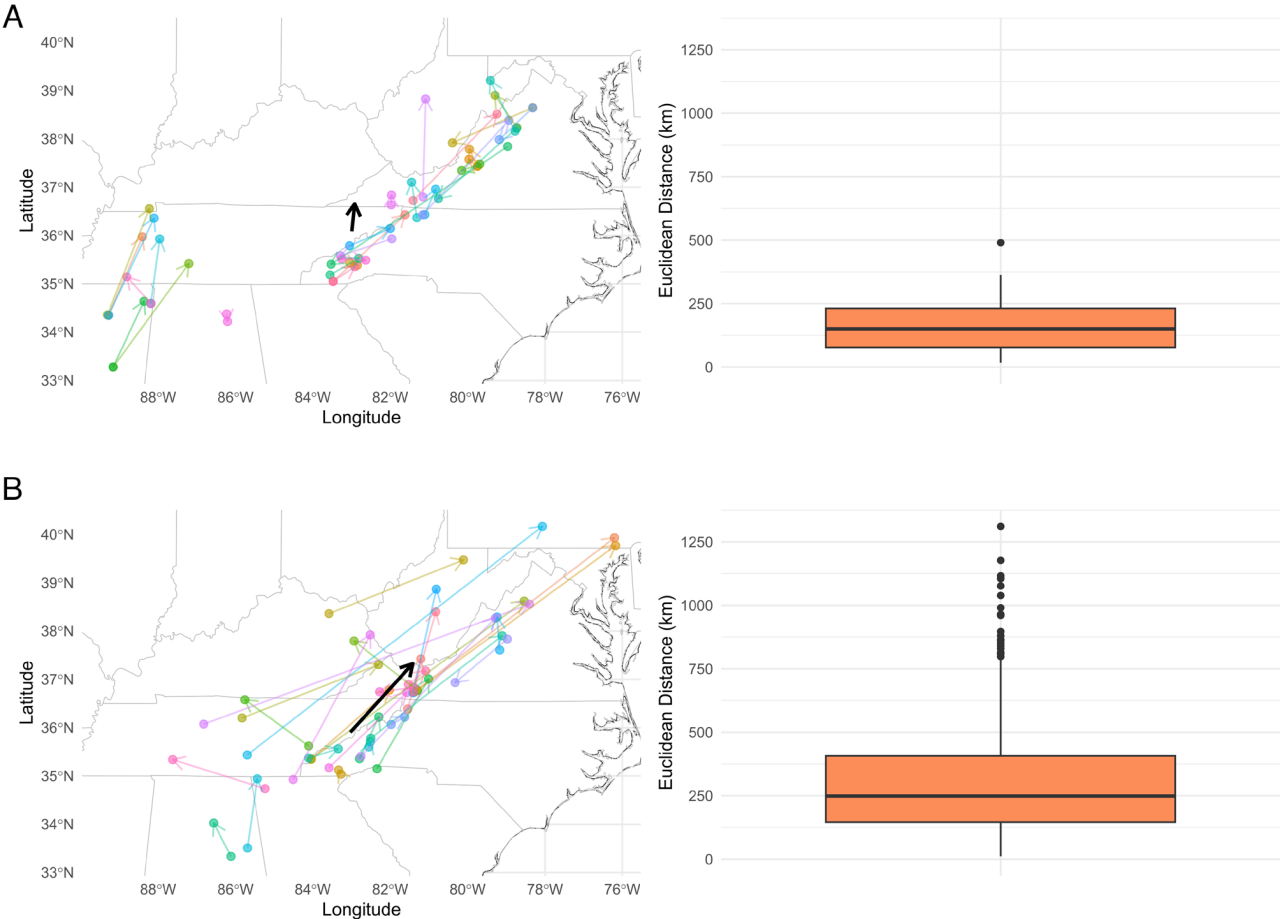
Differences between Locator-predicted origins and known origins. (*A*) Locator-predicted origins of wild trees compared with their actual origin. (*B*) Locator-predicted origins of backcross trees and the actual origin of the most recent wild father. For clarity, only a random subset of 30 samples are shown. In each panel, arrow tails are the known origins and arrow heads are the predicted origins. Bold black arrows represent average deviations across all samples. Boxplots to the right of each panel reflect variation in predicted and actual locations across all samples within each group.

Due to the complex ancestry of the backcross trees, it is difficult to predict from pedigree information alone the geography and climate to which a given tree would be best adapted. We therefore used Locator software and the adaptive SNP dataset to estimate the seed zone to which each tree would be best adapted. Using wild-type trees of known provenance, Locator predicted geographic origin with an average error of 169.82 km (*SI Appendix*, Fig. S13 and Fig. 6). Predicted locations for the backcross trees were also compared to the known location of the most recent wild *C. dentata* parent, and had an average error of 313.16 km (Fig. 6). Assignment of backcross trees to seed zones mostly agreed with the most recent *C. dentata* parent seed zone origin (85.71%).

### 2.7. Breeding Simulations between Backcross and Wild American Chestnut Populations.

We performed breeding simulations to determine whether there would be significant gains in the percentage of adaptive alleles represented and correlations with wild-type AFs from breeding with additional wild-type parents conserved in TACF’s GCOs. For the southwestern seed zone, there was a substantial increase in the correlation between the breeding program and wild-type AFs from breeding backcross trees with additional wild-type trees (r = 0.94) versus intercrossing backcross trees only (r = 0.68). Breeding with additional wild-type trees resulted in a marginal increase in adaptive AF correlations for the central seed zone (backcross + GCO: r = 0.93, backcross only: r = 0.89) and no increases for the northern seed zone (*SI Appendix*, Fig. S14). Doing more intercrosses among backcross trees was predicted to result in small gains in the correlation with wild-type AFs as compared with breeding with additional wild-type trees. For all seed zones, >95% of the allelic diversity was represented with 30 crosses in each seed zone, with marginal gains (<1%) from doing more crosses or breeding with additional wild-type trees (*SI Appendix*, Fig. S14). Overall, the simulations predict that the greatest gains in adaptive diversity can be made by breeding with additional wild-type trees from the southwestern seed zone.

## 3. Discussion

The goals of this study were to describe the genomic basis of local adaptation in American chestnuts, define seed zones for germplasm conservation, develop a quantitative framework for sampling recommendations, and evaluate the adaptive genomic content captured within a backcross breeding program. To do this, we used a SNP dataset arising from high coverage whole-genome resequencing to scan for signatures of environmental adaptation, and to model the partitioning of that adaptive variation across the landscape. These analyses revealed polygenic adaptation to climate that can be most parsimoniously summarized by three seed zones, from which a relatively small number of trees can be sampled to capture 95% of wild adaptive diversity. In addition, despite opportunistic sampling of wild parent trees, the backcross breeding program operated by TACF over the past 40 y has incorporated a reasonable proportion of the wild adaptive diversity into families with improved blight resistance.

### 3.1. Climate Shapes Genomic Variation in American Chestnut.

Like many widespread temperate tree species, climate has significantly shaped genomic variation in American chestnut. Although well connected by gene flow, this species exhibits isolation-by-distance across a natural range with highly variable abiotic environments, in particular precipitation and temperature gradients. We recently showed that the geography of population structure across this range tracks transitions from below freezing winter temperatures in the north to a ≈25% higher annual precipitation in the south (10). Among ≈11.5 million genome-wide SNPs, we identified ≈18.5k associated with climate. Precipitation, its seasonality, and its interaction with temperature in the summer are primary drivers of adaptation in chestnut, comprising the top three and 11 of the top 15 climate variables in the GF analysis. This contrasts with, for example, lodgepole pine and red spruce, for which variables related to winter temperature are most important to adaptation (40, 51). Many of the most studied forest tree species occupy ranges that begin and end further north than that of American chestnut. This dichotomy may reflect a different cold hardiness strategy for chestnut that is insufficient in areas with extremely cold winter temperatures, as well as adaptations to the high summer evaporative demand in the southern portion of the chestnut range.

A limitation of these GEA approaches is that they assume linear relationships between climate and AFs (21, 33). Methods to identify nonlinear relationships between genotypes and environment are available [e.g., GF (23)], but are not computationally efficient as screening tools for large genomic datasets. Modeling nonlinear genotype–environment interactions may be of interest in chestnut and other species, particularly given breaks in temperature and precipitation at population boundaries estimated with genome-wide data, and the correspondence of these breaks to seed zones estimated with our adaptive loci.

### 3.2. Adaptive Units for Germplasm Conservation and Reforestation.

Chestnut restoration efforts will need to account for local adaptation by matching blight resistant families to climatic niches in which they exhibit appropriate timing of growth and dormancy, water use efficiency, drought tolerance, and more. Characterization of the pattern and extent of such local adaptations in forest trees is typically achieved through reciprocal common gardens. As it is not possible to acquire wild seed representative of all or even most climates historically occupied by the species, we sought to use correlations between multivariate genotypes to the climate space in which they are found as a proxy for adaptation. In both scenarios, the concept of seed zones has been an essential tool for land managers to identify unique regions of climate adaptation and to inform candidate areas for reintroduction of seed, often from orchards of known provenance. For chestnut, this matching is further complicated by the hybrid or transgenic nature of the germplasm, which makes provenance a more nebulous concept even if phenotypic local adaptation were well described.

Using both adaptive genomic and climate information, we found that the American chestnut range can be most parsimoniously divided into three seed zones, which best represents the American chestnut adaptive boundaries. These zones roughly match genome-wide population structure (10), which relied on neutral genomic variation to define management units (52, 53). While false positive genotype–environment associations may contribute to this outcome, which suggests our putative adaptive cohort of SNPs may be substantially impacted by false positives that simply recapitulate population structure. We view false positives as an unlikely primary explanation for the synergy between neutral and adaptive variation for four reasons. First, we applied very strict outlier cutoffs, which if anything, biased the dataset toward the exclusion of true positives rather than inclusion of false positives. While the number of adaptive loci in our dataset was very large, it is consistent with the polygenic nature of locally adaptive traits in temperate trees species (9, 54). Second, F_ST_ for the adaptive SNP cohort was 5.5- to 6.5-fold higher than the genome-wide background across the seed zones, suggesting divergent selection. Third, while the three seed zones roughly track background population structure, there were important differences. For example, SZ.1 extended southward through high elevation areas of the state of West Virginia, which is not reflected in the neutral population structure map, and there was an extension of SZ.2 southward and SZ.3 northward along the border of Tennessee and North Carolina, which reflects a demarcation between the Great Smoky Mountains in the east (SZ.2) and the much warmer Tennessee Valley to the west (SZ.3). Finally, and more generally, in a species for which the axes of postglacial recolonization and climate differentiation are aligned, population structure and adaptation will tend to parallel each other, and this effect will be apparently exaggerated by collapsing high dimensional adaptive diversity data into a small number of clusters. Nevertheless, such summaries facilitate the incorporation of adaptive diversity information into operational breeding and reintroduction strategies that entail multiple competing priorities.

### 3.3. Matching Adaptive Portfolios to Future Climates.

American chestnut has clearly adapted to regional precipitation and temperature regimes, and while the above seed zones provide a spatial delineation of how genotypes match geography, these estimates reflect historical climate. Under climate change, the coupling of climate and geography in the context of our modeling will break down, and matching genotypes to planting sites will require reconciling current climates that interact with seedling fitness, and future climates that impinge on competitive ability, reproduction, recruitment, and adaptation of subsequent generations. We found that the central portion of the chestnut range, primarily to the east and west of the Appalachian Mountains, will experience more pronounced maladaptation. Conversely, the southern portion of the range is projected to experience milder maladaptation. This result is somewhat counterintuitive in that southern trailing edge populations (in the northern hemisphere) tend to inhabit the highest temperature environment for a given species range, and should therefore be most vulnerable to increases in temperature. However, temperature may not be the primary constraint in all cases. For example, Gougherty et al. (55) found that longitudinal edge populations of the *Populus balsamifera*, rather than those at the southern trailing edges, will experience the highest offset, which was due to the importance of winter precipitation. For chestnut, these southern locations are highly diverse, both genome-wide and for genotype–environment associated SNPs, so may be to a certain degree preadapted to higher temperatures.

It is important to note that our genomic offset analysis focuses on regions within the historical range of the American chestnut. This focuses our interpretations on the impacts of evolving climate conditions on existing stands, given the lack of natural migration capacity for this species. As a complementary approach, the offset analysis allows us to target future plantings of specific genetic material to future climates. In general, this analysis suggests moving material ~400 km to the northeast of the location for which it would have been best adapted historically. It should be noted that particular locations representing a climate match for a given seed zone are not necessarily suitable for chestnut, as soil conditions and biotic factors were not considered. Conversely, locations that do not contain matching pixels are not necessarily unsuitable for replanting, but rather did not minimize genomic offset. As such, Fig. 4*B* should be viewed as a suggestion for an average range shift north for each seed zone for replanting efforts and should be combined with additional geographic and ecological data to circumscribe possible planting locations for planting of germplasm with a particular adaptive genomic composition.

A logical next step will be to extrapolate our model to identify areas outside of the current species range for the targeting of restoration plantings. Climate change will likely shift the habitable range of American chestnut northward (56, 57), thus areas farther north may be considered for reintroduction. While the concept of assisted migration does not exactly fit the case of American chestnut—a species for which there are no natural populations to migrate—it can nevertheless provide a framework in which to think about the differential targeting of locally adapted restoration populations to future geography and climate (58). For example, chestnuts planted northward outside of the historical range may suffer winter injury, but exhibit increased combined growth and survival ordinal scores than most other assisted migration tree species from the eastern United States (red spruce, northern red oak, eastern hemlock, black cherry, bigtooth aspen, black birch, and bitternut hickory) (59). Germplasm from the central and northern seed zones (SZ.2 and SZ.1, respectively), exhibit less winter injury and should be considered for use in the most northern replanting locations (13). We recommend additional evaluations of the three seed zones identified here in common garden and controlled environment experiments to determine how germplasm from each of these zones will respond to assisted migration and future climate conditions. This additional information will ensure that locally adapted American chestnut trees will be replanted in regions where they are adapted.

### 3.4. Sampling Estimates for Germplasm Conservation of Wild Adaptive Genomic Diversity.

Ultimately, we sought to estimate the number of trees to sample from each seed zone to capture most of the adaptive diversity present in the wild. These sampled trees will be used for germplasm conservation, and to introgress adaptive variation into backcross and genetically engineered populations. Consistent across different thresholds for sufficient capture of adaptive diversity, we found that the fewest trees will need to be sampled from the southern SZ.3, and the most from the northern SZ.1. These findings reflect that genomic diversity is highest in the southern chestnut range and decreases as latitude increases (10, 60–62). Thus, fewer trees would need to be sampled from regions of higher heterozygosity and nucleotide diversity to capture the desired percentage of adaptive diversity.

### 3.5. Adaptive Genomic Diversity Captured Within the TACF Backcross Breeding Program.

Beginning in the late 1980s, TACF’s backcross breeding program aimed to develop disease-resistant American × Chinese chestnut hybrids that retain the disease-resistant traits of the Chinese chestnut, but have the competitive forest tree growth form of American chestnut. Presently, selected hybrids from TACF’s breeding program have resistance intermediate between Chinese chestnut and American chestnut, while also inheriting between 60% and 95% of their genome from American chestnut. Further generations of intercrossing and selection will be necessary to enhance blight resistance. The process of developing an enhanced disease-resistant tree involved several generations of backcrossing the interspecific F1 hybrids with pollen from wild American chestnut father trees (4). While pollen used in backcrossing was sourced from rare, wild, flowering trees, we sought to understand whether this haphazard sampling of wild diversity has sufficiently represented the adaptive genomic content of the wild American chestnut range.

Our results suggest that the backcross breeding strategy has in fact captured much of this wild adaptive genomic diversity. However, two aspects of our results warrant further consideration. First, we observed some residual variance (≈20%) in the adaptive AF distribution between wild and backcross groups. Part of this was due to *C. mollissima* ancestry, an inevitable consequence of its hybrid origins and of the hitchhiking effect that is a consequence of selection for blight resistance. But this was also driven by a bias toward medium-frequency alleles in the wild and rare alleles in the backcross group, and the potential for fixation of these low-frequency alleles may need to be considered in future selections and crosses. Second, only ≈8.8% of the adaptive ancestry was associated with SZ.3. This seed zone is located in the southern region of the historical chestnut range – which was also the region with the highest overall genomic diversity (10, 60, 62). Future efforts to sample adaptive diversity through grafting and pollen collection should pay special focus to this underrepresented region, as it represents a reservoir of historical diversity that has been less affected by cyclical bottlenecks associated with Quaternary glaciation, but more specifically because it is the warmest part of the contemporary range, and likely comprises unique alleles that will be important under climate change (63). While SZ.3 has lower numbers of trees recommended to be sampled compared with the other seed zones, the greatest gains in adaptive diversity can be made through additional breeding with SZ.3 wild-type parents. Thus, it would be prudent to err on the side of caution and oversample SZ.3 when developing a breeding plan for diversifying the backcross (and transgenic) populations.

### 3.6. Replanting Locations Can be Predicted for Backcross Trees.

The backcross population contains trees with diverse ancestry, with most arising from at least two seed zones and overall representing many pollen parents. As such, determining where these trees are likely to be best adapted is a challenge. The Locator tool was designed to infer the original location of plant and animal samples using SNP data and a training set of individuals with known locations (45). Our use of Locator with a putative adaptive SNP dataset should provide estimates of a tree’s origin based on their local adaptation—leading to an estimate of the geographic origin of each tree’s multilocus adaptive genotype, which can serve as a proxy for the best fit replanting environment. The performance of Locator for wild trees of known provenance was very high, which suggests it reliably integrates the adaptive SNP information with respect to geography. In the case of backcross trees, we cannot train a model that precisely reflects geographic reality given the complex history of these trees, and the fact that the link to geography is not known for much of this ancestry. Coupled with the polygenic nature of climate adaptation, this led to less agreement between “known” and predicted origins for the backcross trees, where known only refers to the last pollen parent. As such, we suggest that Locator results take precedence when targeting backcross trees to planting environments, with due consideration for additional shifts informed by genomic offset.

### 3.7. Conclusions.

American chestnut is a functionally extinct species facing both biotic and abiotic threats. Developing disease-resistant populations is an essential first step to restoration of the species, but understanding adaptive variation in the context of a wide geographic and climatic historical range is equally critical to restoration. However, little was previously known about local adaptation in this species. Our goal was to develop a parsimonious strategy for conserving and deploying the reservoir of wild adaptive diversity available in the form of root collar sprouts that predate the introduction of chestnut blight. We have shown that this objective can be advanced with relatively modest investment in sampling, grafting, and collection of pollen from each of three adaptive or “seed” zones (although wild chestnut seed is exceptionally rare). Another important outcome of this work is the finding that historical collections of pollen in the wild, the genetics of which was incorporated into TACF’s breeding program, has effectively captured much of this diversity already. However, while most adaptive SNPs segregate in these populations, we found that the minor allele was more commonly rare than in the wild, which means that careful attention to adaptive variation through future breeding cycles will be needed so as not to lose this variation downstream. In addition, the backcross breeding program was somewhat deficient in genomic variation relevant to the southern seed zone, which will likely be important under climate change, and special efforts must be made to conserve that diversity from wild trees. In the future, the outcomes of our research should be leveraged in the form of common garden studies that link adaptive traits with adaptive genomic variation. As it is impossible to collect sufficient wild seed for comprehensive common gardens, our results provide a unique opportunity to understand trait reaction norms underlying rangewide climatic variation through “adaptive georeferencing” of germplasm with complex ancestry by coupling our comprehensive suite of adaptive SNPs with modern statistical population genetic approaches.

## Supplementary Material

Appendix 01 (PDF)

## Data Availability

Code: The script for the Python sampling method can be found at https://github.com/alex-sandercock/Capturing_genomic_diversity (64). R package version of the Python sampling method can be found at https://github.com/alex-sandercock/castgen (65). The R code used to estimate the seed zone shifts due to climate change is available at Zenodo: https://zenodo.org/doi/10.5281/zenodo.10676843 (66). Genomic Data: The genomic data for the TACF backcross and wild American chestnut samples were submitted to NCBI SRA BioProject PRJNA1070245 (67). The climate adaptive VCF file for the wild American chestnut samples is available at Zenodo: https://zenodo.org/doi/10.5281/zenodo.10676843 (66). Seed Zone files: The shape files and raster files for the wild Seed Zones are available at Zenodo: https://zenodo.org/doi/10.5281/zenodo.10676843 (66). Previously published data were used for this work (10).
