# Supplementary material for: A genome-guided strategy for climate resilience in American chestnut restoration populations
Source: Proc Natl Acad Sci U S A. 2024 Jul 16;121(30):e2403505121. doi: 10.1073/pnas.2403505121 (PMC11287244; doi:10.1073/pnas.2403505121)
Supplement: Supplementary file 1 — Appendix 01 (PDF) [file pnas.2403505121.sapp.pdf]

PNAS

Supporting Information for

A genome guided strategy for climate resilience in American chestnut restoration populations

Alexander M Sandercock[1,2], Jared W Westbrook[3], Qian Zhang[4], and Jason A Holliday[4*]

[1]Genetics, Bioinformatics, and Computational Biology, Virginia Tech, Blacksburg, VA, 24060

[2]Breeding Insight, Cornell University, Ithaca, NY, 14853 [current affiliation]

[3]The American Chestnut Foundation, Asheville, NC, 28804

[4]Department of Forest Resources and Environmental Conservation, Virginia Tech, Blacksburg, VA, 24060

* Corresponding author: Jason A Holliday

Email: jah1@vt.edu

This PDF file includes:

Figures S1 to S15
Tables S1 to S6

Table S1. The 32 climate variables from ClimateNA that were used for this study and their respective descriptions.

Abbreviation	Description
Annual variables directly calculated from monthly variables	
MAT	mean annual temperature (°C)
MWMT	mean warmest month temperature (°C)
MCMT	mean coldest month temperature (°C)
TD	temperature difference between MWMT and MCMT, or continentality (°C)
MAP	mean annual precipitation (mm)
AHM	annual heat-moisture index ((MAT+10)/(MAP/1000))
SHM	summer heat-moisture index ((MWMT)/(MSP/1000))
Annual variables derived from monthly variables	
DD_0	degree-days below 0°C, chilling degree-days
DD5	degree-days above 5°C, growing degree-days
DD_18	degree-days below 18°C, cooling degree-days
DD18	degree-days above 18°C, heating degree-days
NFFD	the number of frost-free days
FFP	frost-free period
bFFP	the day of the year on which FFP begins
eFFP	the day of the year on which FFP ends
PAS	precipitation as snow (mm). For individual years, it covers the period between August in the previous year and July in the current year
EMT	extreme minimum temperature over 30 years (°C)
EXT	extreme maximum temperature over 30 years (°C)
Eref	Hargreaves reference evaporation (mm)
CMD	Hargreaves climatic moisture deficit (mm)
MAR	mean annual solar radiation (MJ m-2 d-1)
RH	mean annual relative humidity (%)
CMI	Hogg’s climate moisture index (mm)
DD1040	degree-days above 10°C and below 40°C
Seasonal variables directly calculated from monthly variables	
Tave_wt	winter mean temperature (°C)
Tave_sp	spring mean temperature (°C)
Tave_sm	summer mean temperature (°C)
Tave_at	autumn mean temperature (°C)
PPT_wt	winter precipitation (mm)
PPT_sp	spring precipitation (mm)
PPT_sm	summer precipitation (mm)
PPT_at	autumn precipitation (mm)

Table S2. The environmental variable loadings on the three PC axes used for the LFMM analysis.

	PC1	PC2	PC3
RH	-0.048	-0.034	0.937
Eref	0.378	0.324	-0.111
PPT_wt	0.416	-0.116	0.053
EXT	0.039	0.472	-0.073
TD	-0.437	0.01	-0.134
MAR	0.443	0.006	0.027
PPT_sm	0.351	-0.256	-0.156
CMI	0.132	-0.498	0.111
AHM	-0.119	0.487	0.156
DD_18	-0.373	-0.326	-0.148

Table S3. Number of single-nucleotide polymorphisms (SNPs) in coding and noncoding features. Three datasets were evaluated: **Adaptive** (18,483 putatively adaptive SNPs from the GEA analyses), **Random** (randomly selected SNPs with allele frequency distribution matched to the adaptive set), and **Full** (original filtered SNP dataset from Sandercock et al. (10) with MAF<0.05 filter). The promoter region includes the 2kb region upstream of the mRNA intervals. Percentage of the total SNPs for each feature are shown in parentheses.

	Adaptive	Random	Full
Number of SNPs in CDS	264 (1.43%)	619 (3.35%)	377161 (3.27%)
Number of SNPs in exon	435 (2.35%)	991 (5.36%)	611402 (5.30%)
Number of SNPs in five_prime_UTR	69 (0.37%)	141 (0.76%)	89577 (0.78%)
Number of SNPs in three_prime_UTR	104 (0.56%)	236 (1.28%)	150477 (1.31%)
Number of SNPs in mRNA	2120 (11.47%)	3462 (18.73%)	2102039 (18.24%)
Number of SNPs in promoter	1309 (7.08%)	1830 (9.90%)	1154261 (10.01%)
Number of SNPs in gene	2120 (11.47%)	3462 (18.73%)	2102039 (18.24%)
Number of SNPs in intron	1712 (9.26%)	2537 (13.73%)	1528332 (13.26%)
Number of synonymous SNPs	113 (0.61%)	227 (1.23%)	147714 (1.28%)
Number of non-synonymous SNPs	132 (0.71%)	336 (1.82%)	190153 (1.65%)
Number of non-coding SNPs	18238 (98.67%)	17921 (96.96%)	11189046 (97.10%)
Total SNPs in dataset	18483	18483	11526713

Table S4. Number of putatively adaptive genes containing at least one adaptive SNP within each *Castanea dentata* chromosome. Chr06, Chr10, and Chr12 contained significantly more adaptive SNPs than expected under the null of even distribution relative to the number of tested SNPs per chromosome (P ~ 0 based on exact binomial test in R). Scaffolds without genes containing adaptive loci were omitted.

Chromosome	Adaptive genes	Total genes	Expected adaptive genes	p-value
Chr01	53	4334	60	0.835
Chr02	19	2599	36	0.999
Chr03	20	2505	35	0.998
Chr04	33	2199	31	0.383
Chr05	7	2983	41	1.000
Chr06	71	2913	40	≤ 0.001
Chr07	28	2095	29	0.600
Chr08	18	2166	30	0.993
Chr09	42	2163	30	0.021
Chr10	68	2120	29	≤ 0.001
Chr11	7	2753	38	1.000
Chr12	66	2260	31	≤ 0.001

Table S5. Estimated number of samples to capture adaptive diversity from each seed zone for a two seed zone model.

Seed zone	% variance explained	# of trees to sample	95% CI
1	90%	16.73	(15.69, 17.77)
2	90%	11.05	(10.13, 11.97)
1	95%	34.12	(31.90, 36.34)
2	95%	21.91	(20.11, 23.71)
1	99%	167.56	(156.37, 178.75)
2	99%	107.37	(100.20, 114.54)

Table S6. Estimated number of samples to capture adaptive diversity under a single seed zone model. The estimate for the 99% variance explained did not complete due to excessive run time.

Seed zone	% variance explained	# of trees to sample	95% CI
1	90%	26.86	(24.55, 29.17)
1	95%	55.89	(51.01, 60.77)
1	99%	DNF	DNF

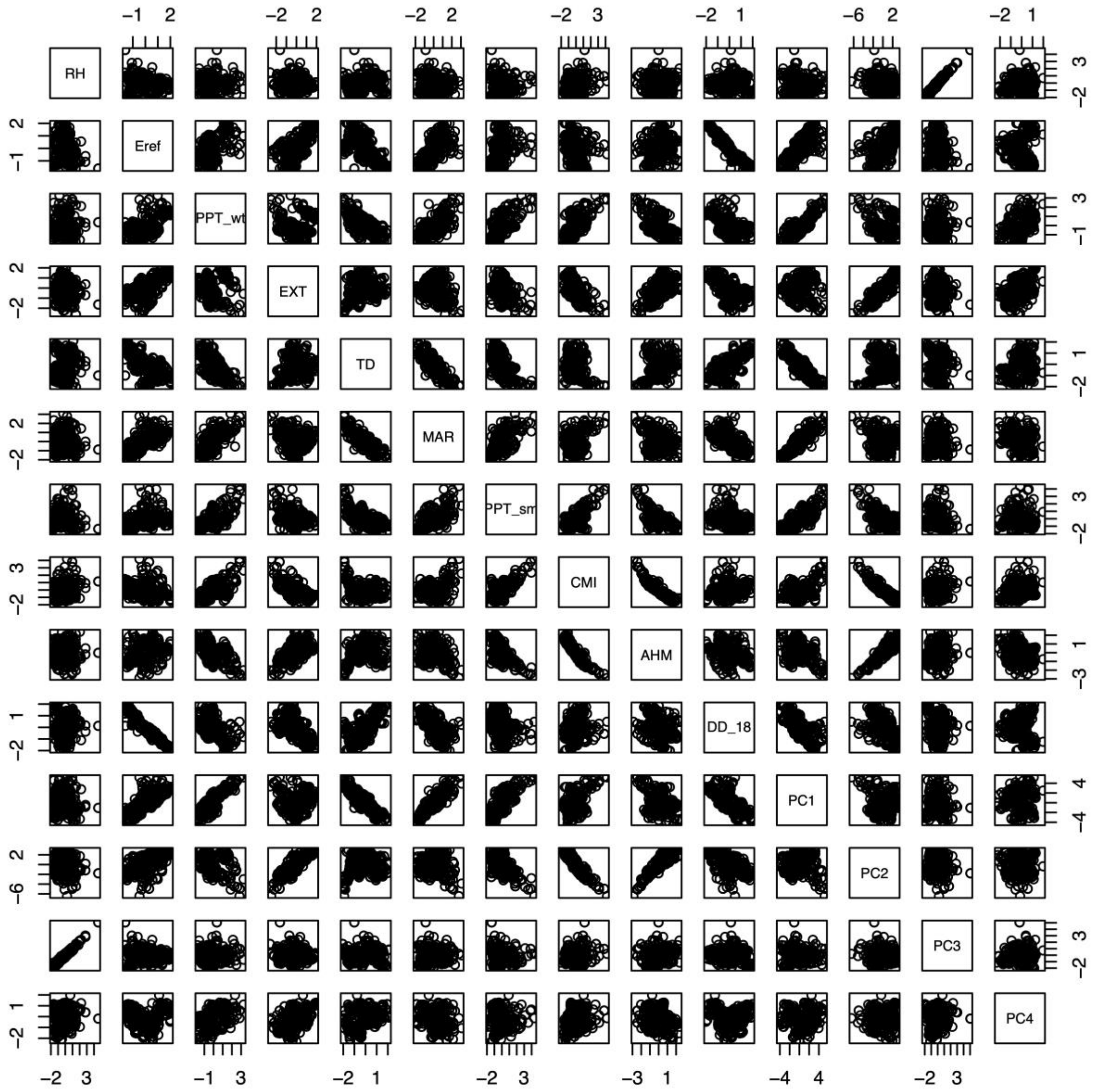

Fig. S1. Three PC axes were used as synthetic variables for the LFMM genotype environment association analyses. Pairs plot of the selected 10 environmental variables and the first four PCs from the PCA of the 10 climate variables. The bottom half and top half are linear regressions between the variables.

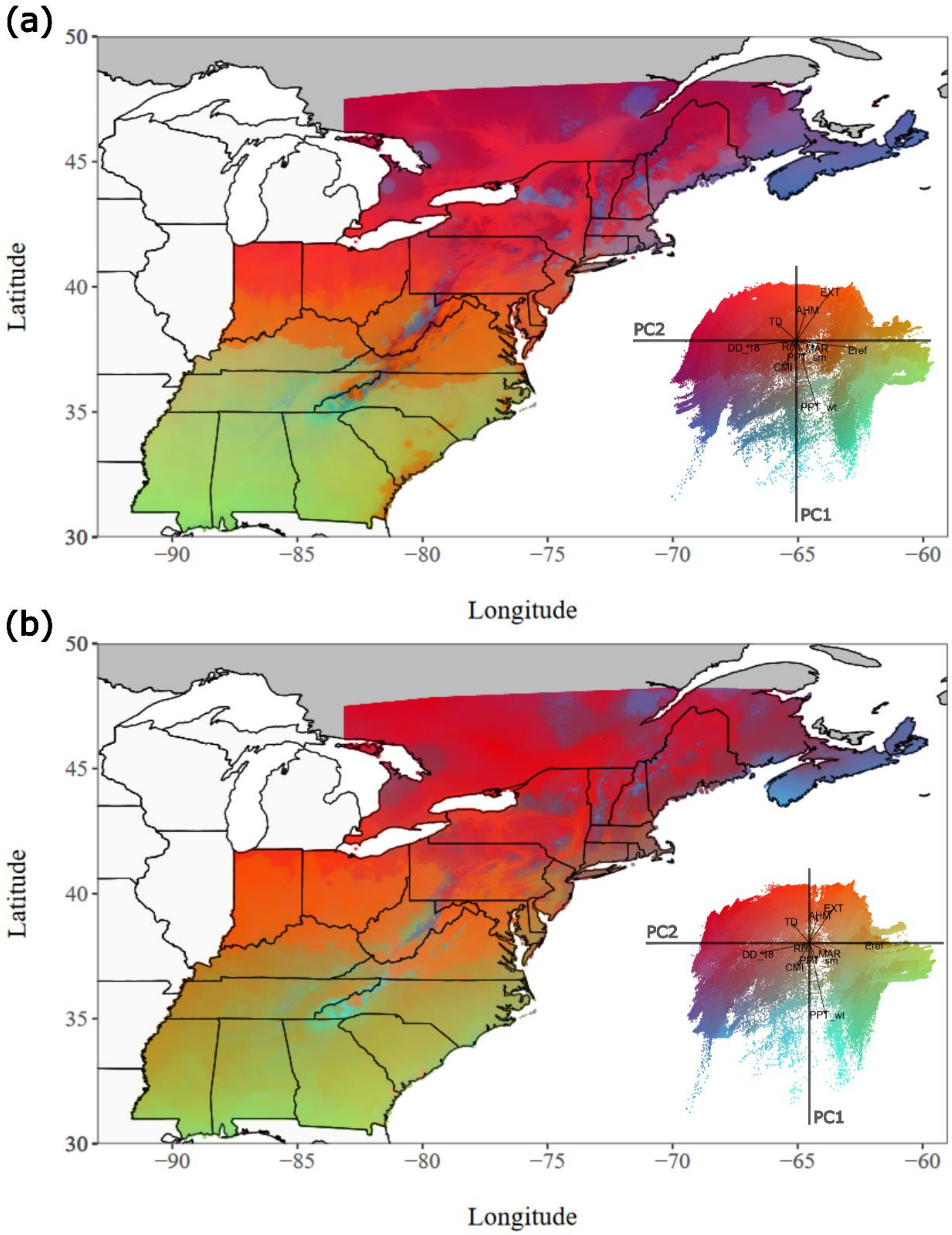

Fig. S2. Projected spatial distribution of genomic composition for future 2080 climate predictions. PC loadings from the gradientForest model of 10 uncorrelated climate variables and 18,483 putatively adaptive loci for the (a) 2080 moderate emissions scenario RCP 4.5 and (b) 2080 severe emissions scenario RCP 8.5. (Inset a,b) Biplot of loadings for PC1 and PC2. These spatial distributions of genomic composition for the 2080 climate predictions were used to estimate the seed zone range shifts necessary to minimize genomic offset.

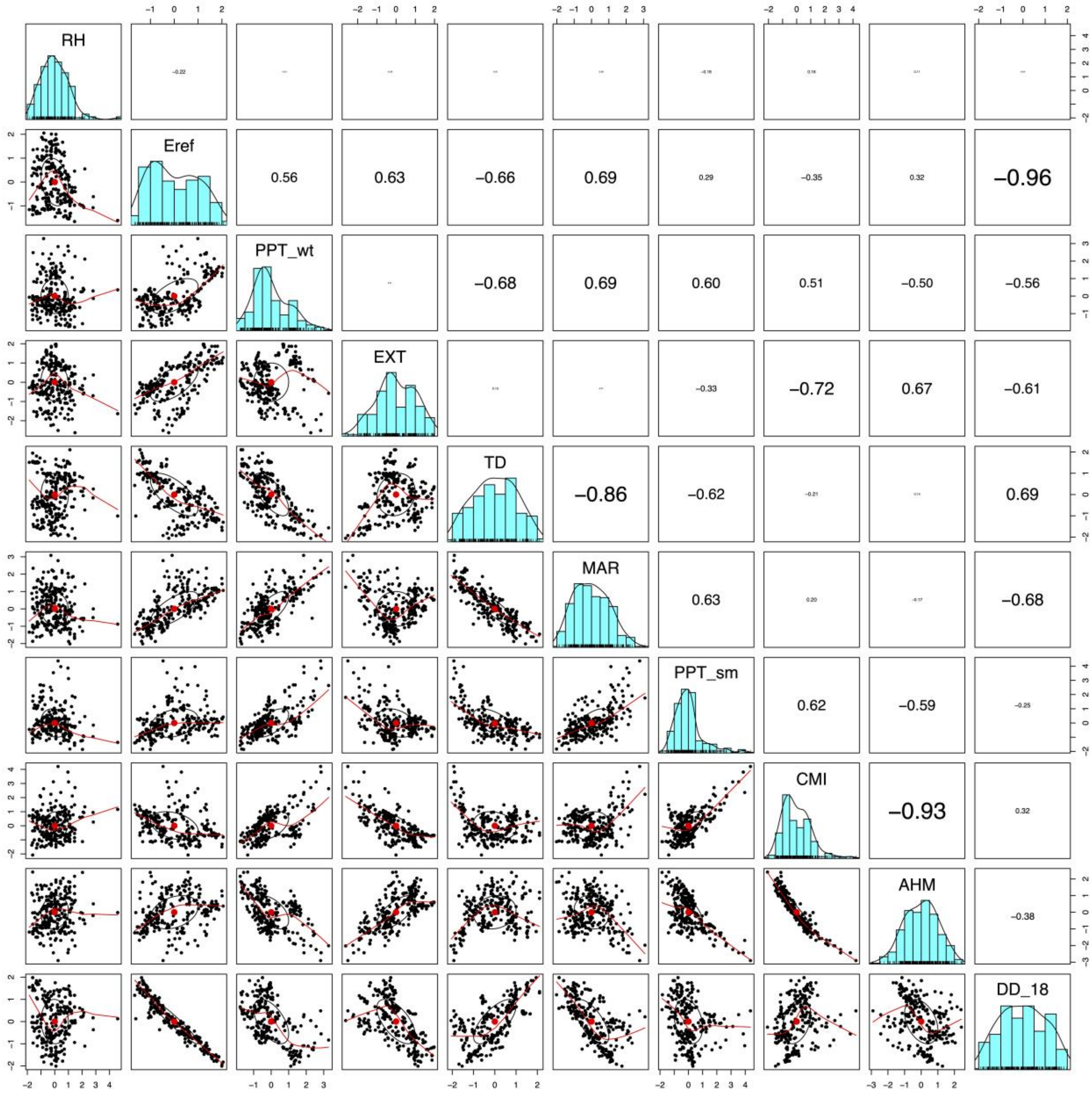

Fig. S3. Ten uncorrelated climate variables were selected for use in the genotype-environment association analyses. Pairs plot of the selected 10 environmental variables. The bottom half are regression plots and the top half are Pearson's correlations.

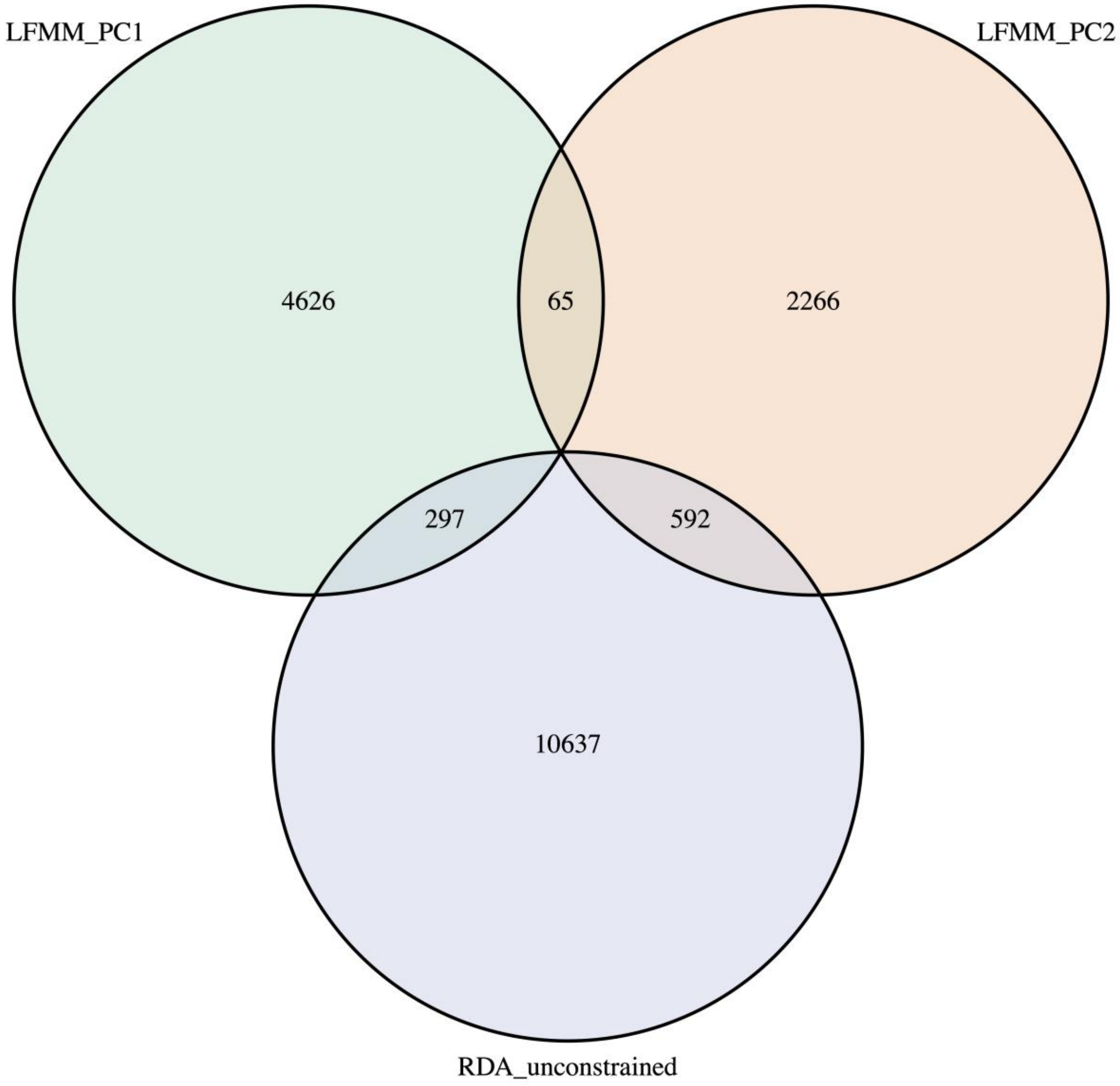

Fig. S4. Venn diagram for the 18,483 adaptive loci identified using RDA and LFMM. For LFMM, only the SNPs from the PC1 and PC2 analyses are shown due to the PC3 analysis finding zero outlier loci.

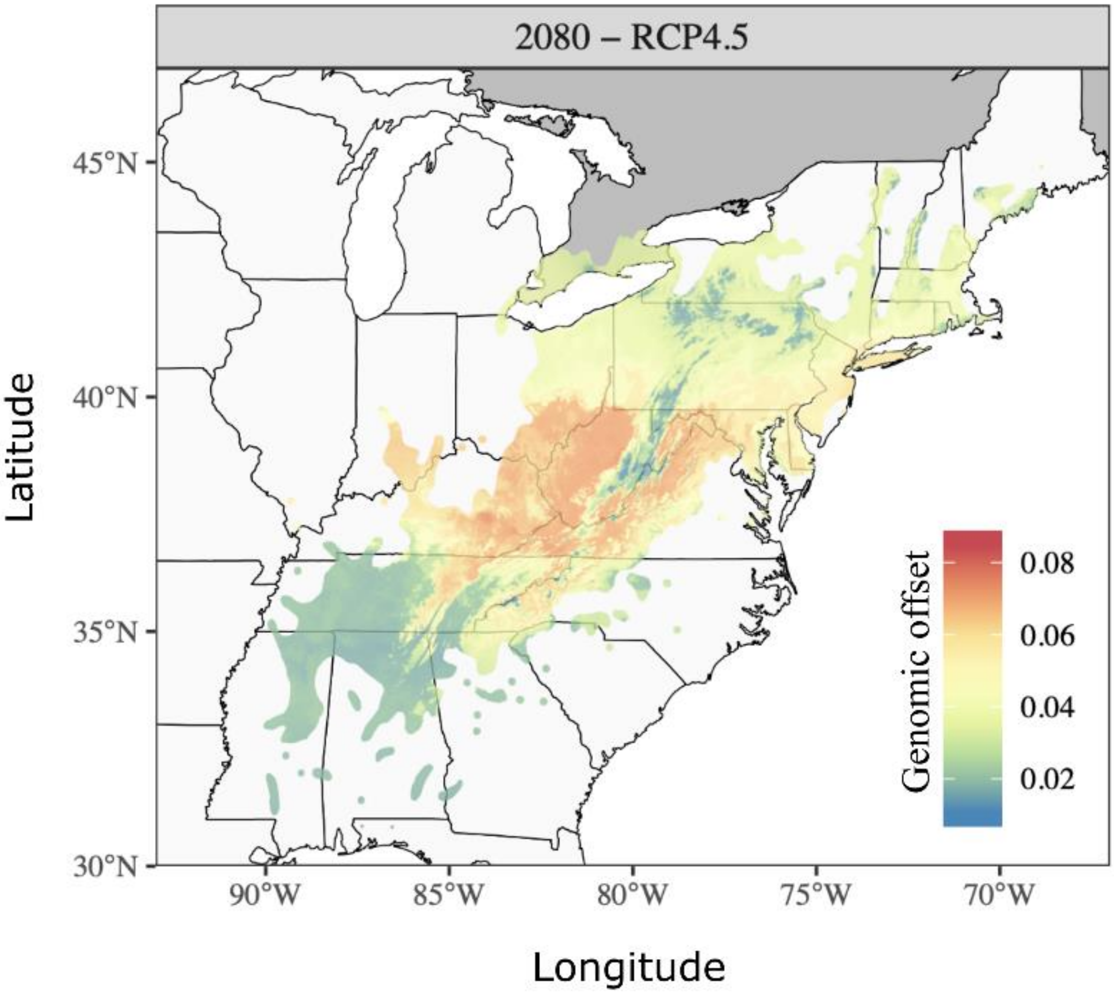

Fig. S5. Projected impacts of climate change throughout the American chestnut natural range. Genomic offset under 2080 climate projections for moderate emissions (RCP4.5).

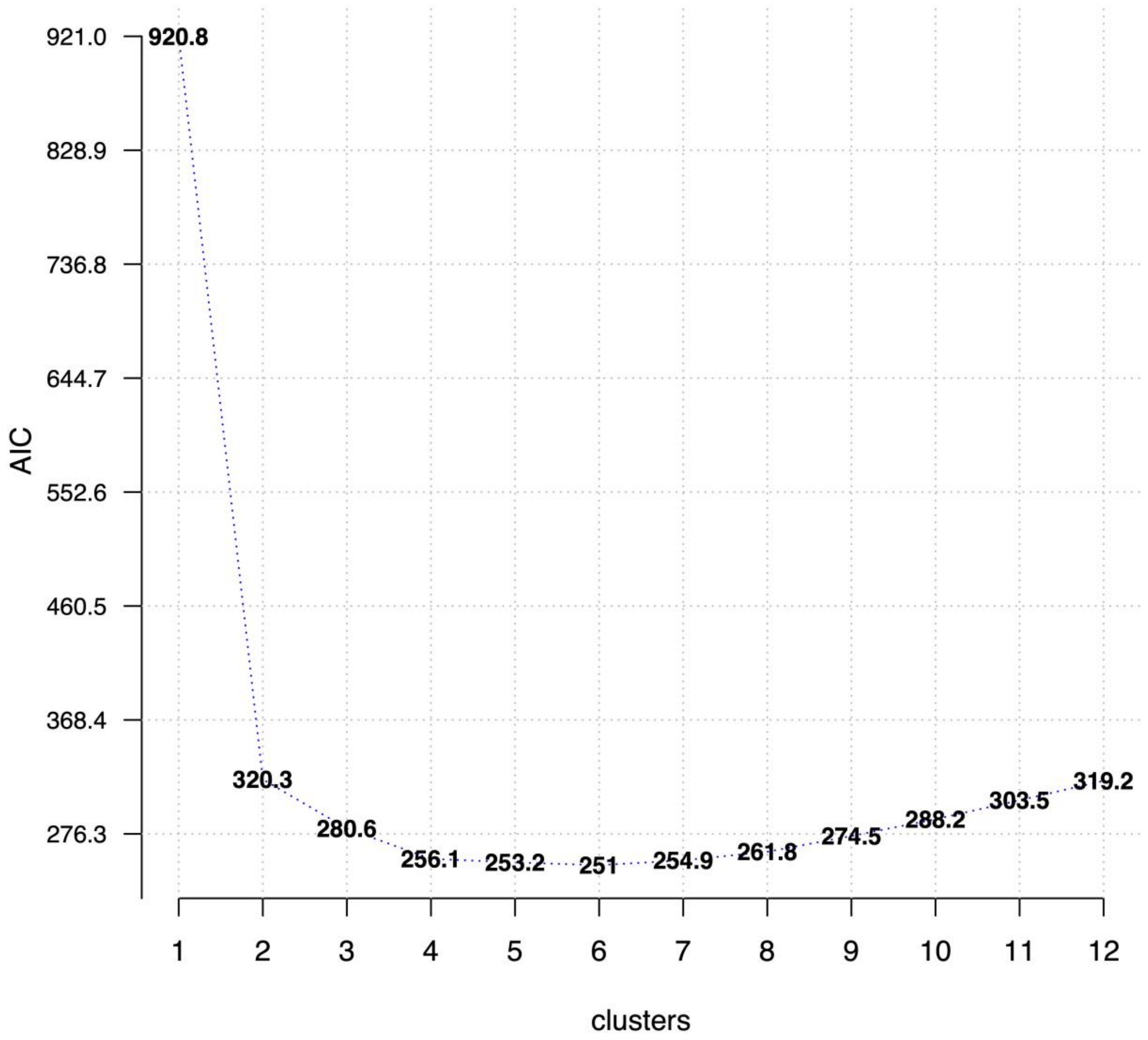

Fig. S6. AIC score evaluation to determine optimal number of seed zones.

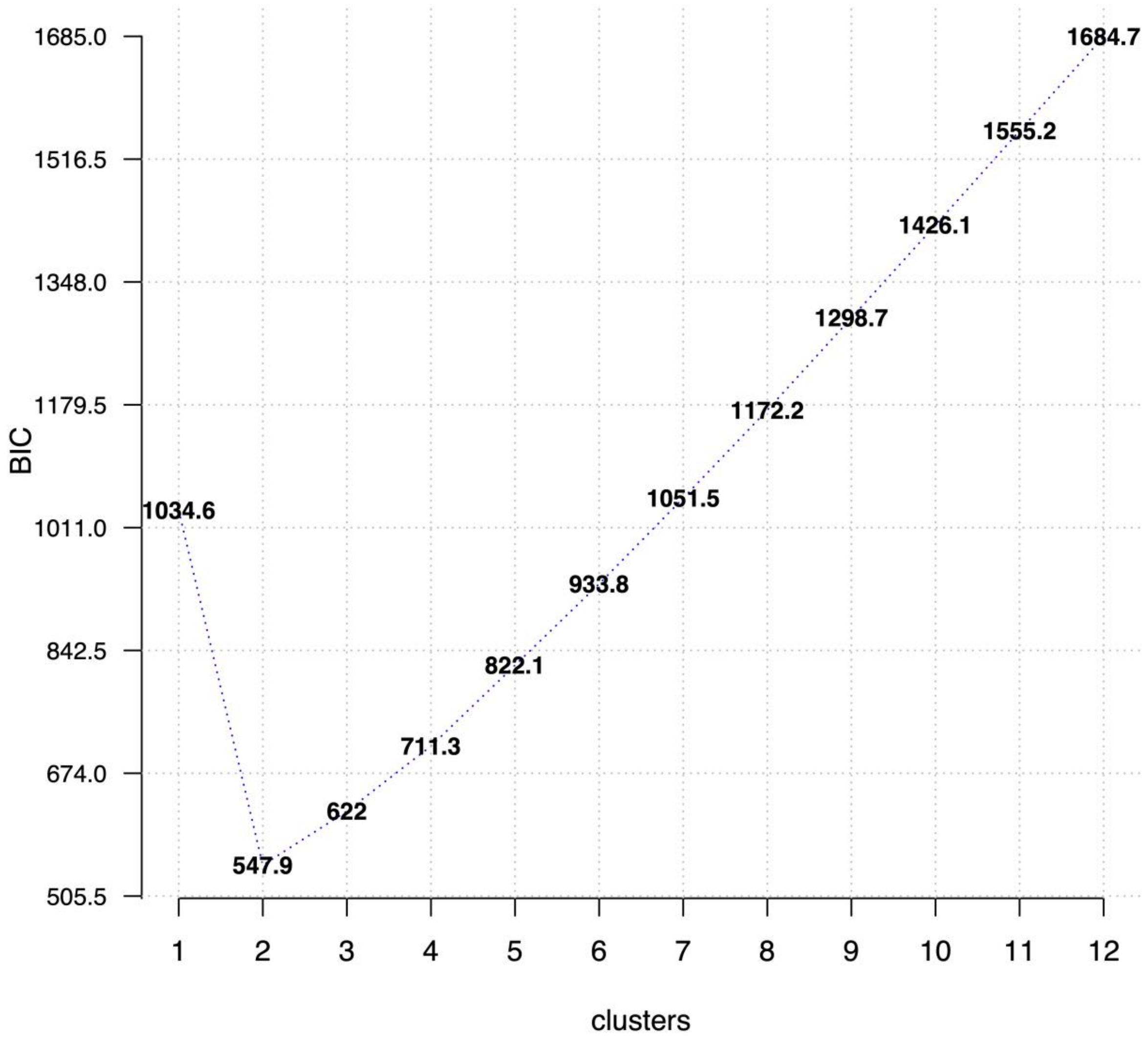

Fig. S7. BIC score evaluation to determine optimal number of seed zones.

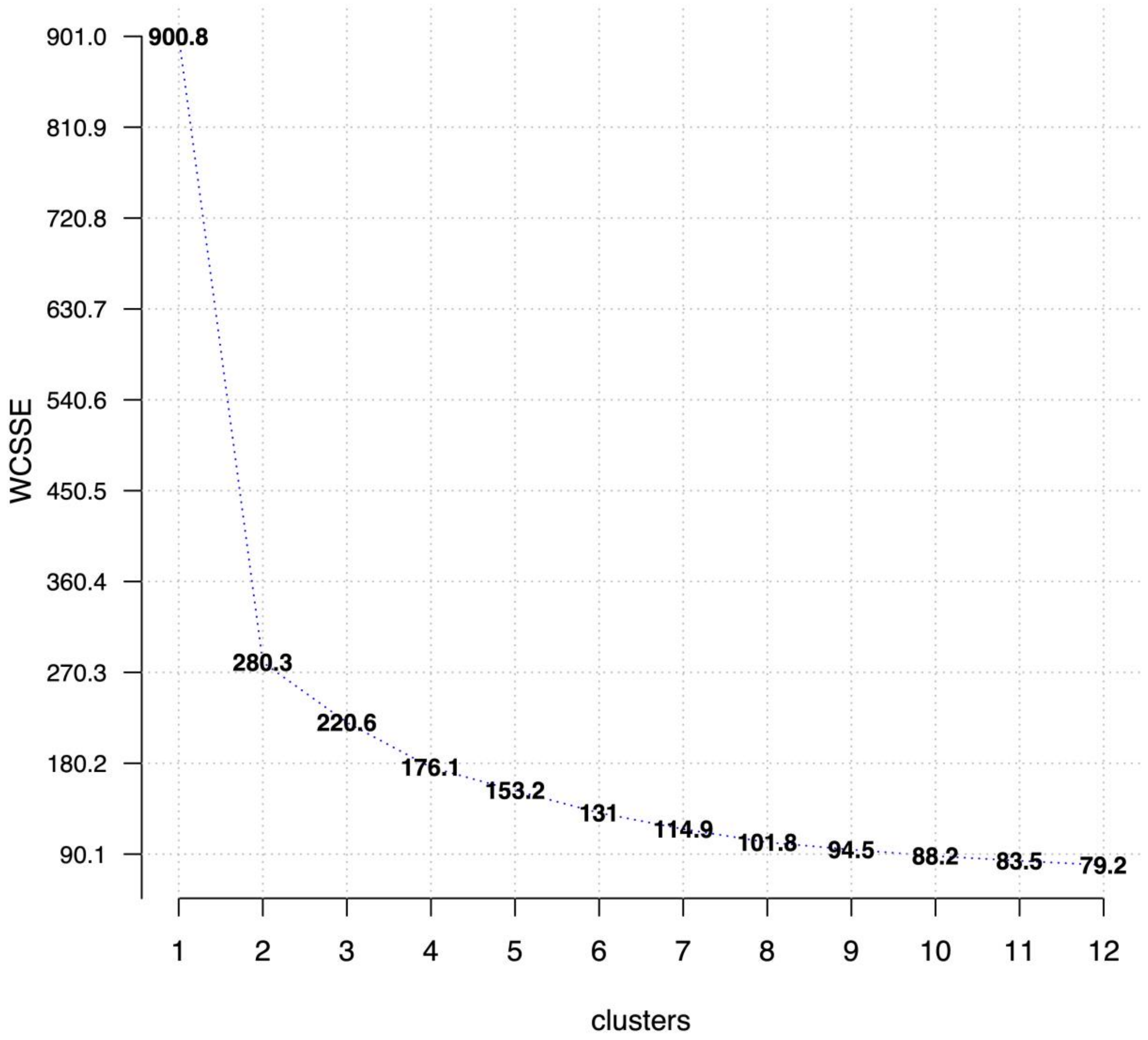

Fig. S8. WCSSE score evaluation to determine optimal number of seed zones.

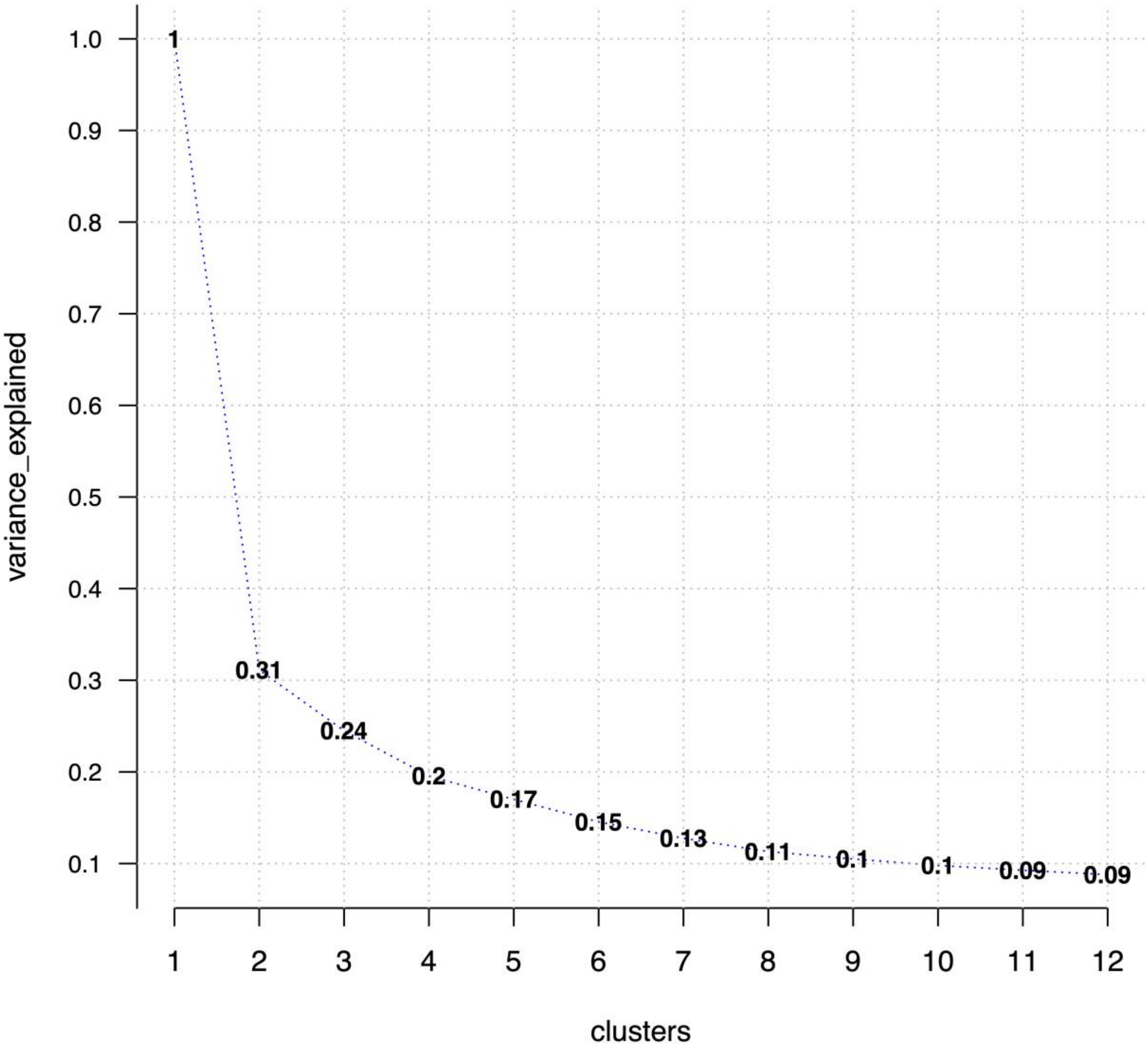

Fig. S9. Variance explained scores to determine optimal number of seed zones.

	Seed Zone 1	Seed Zone 2	Seed Zone 3
Seed Zone 1		0.09633	0.46035
Seed Zone 2	0.01632	ADAPTIVE F_{ST} / NEUTRAL F_{ST}	0.35895
Seed Zone 3	0.08213	0.05540	

Fig. S10. F_{ST} values between each seed zone at randomly selected neutral loci (left) and putatively adaptive loci (right). Approximately 5.5-6.5 fold higher at climate-associated loci compared with neutral loci.

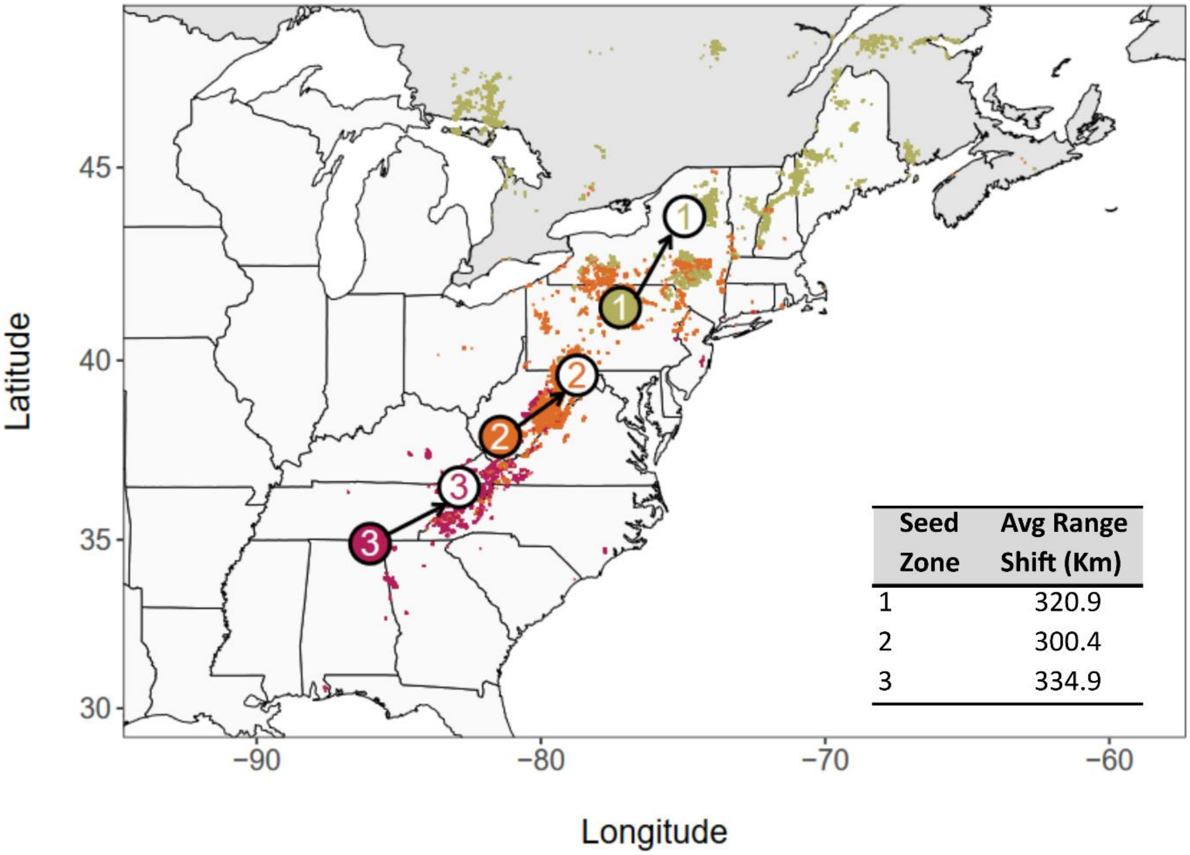

Fig. S11. Projected shifts in seed zones under climate predictions for 2080 RCP 4.5. The solid-colored numbers are the centroids of the historical seed zones, while the white background numbers are the projected shifts of the centroids for the 2080 RCP 8.5 climate projections. The colored pixels are the locations where the genomic offset was lowest for each seed zone under the future climate predictions (red = Seed Zone 3, orange = Seed Zone 2, green = Seed Zone 1). Inset: The distance in kilometers between the historical and projected centroids for each seed zone.

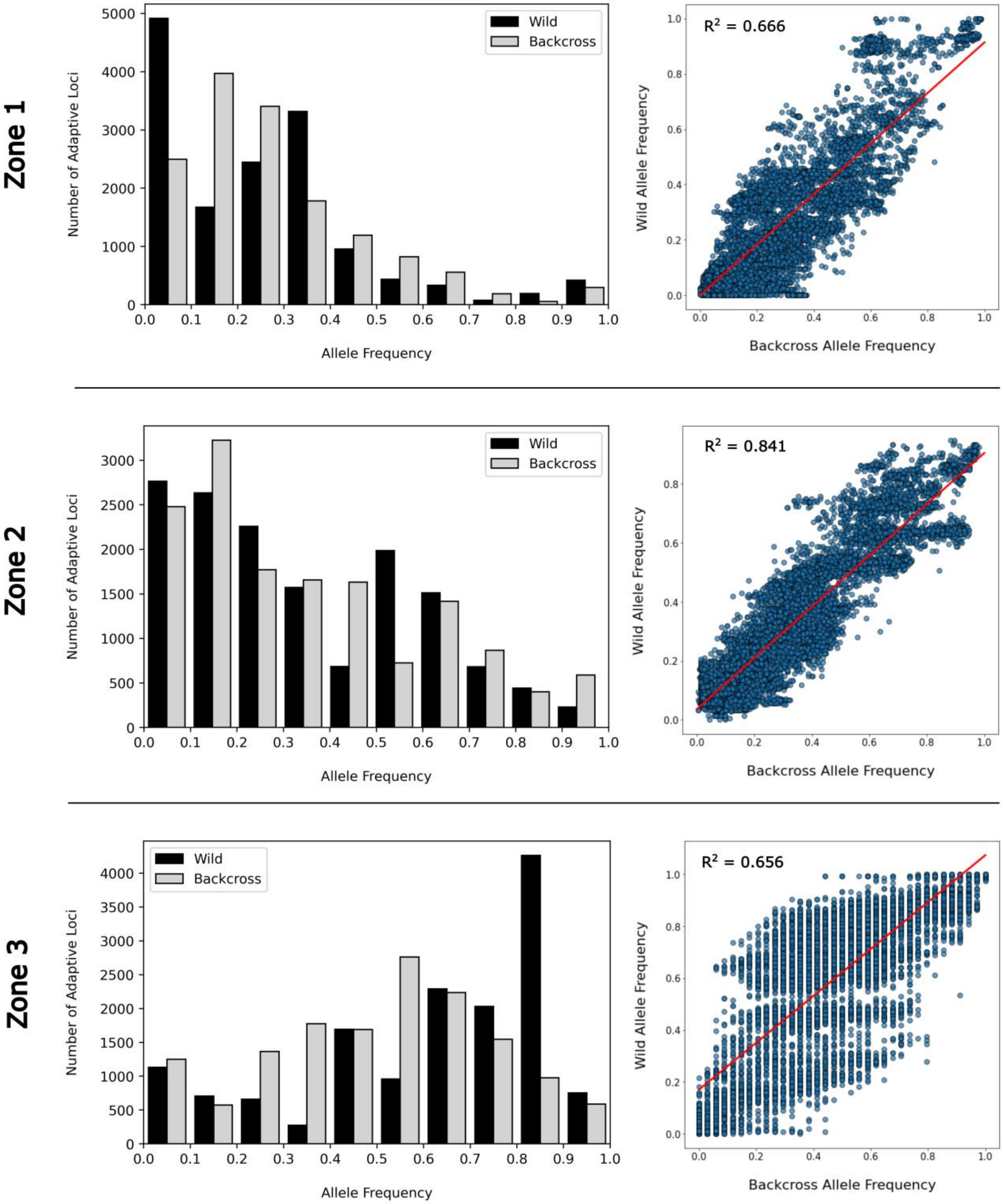

Fig. S12. Comparisons of the allele frequencies for the shared adaptive loci in wild and backcross seed zones. (Left) Allele-frequency histogram at shared adaptive loci for the wild and backcross populations. (Right) Linear regression plot of allele frequencies at shared adaptive loci for the wild and backcross populations.

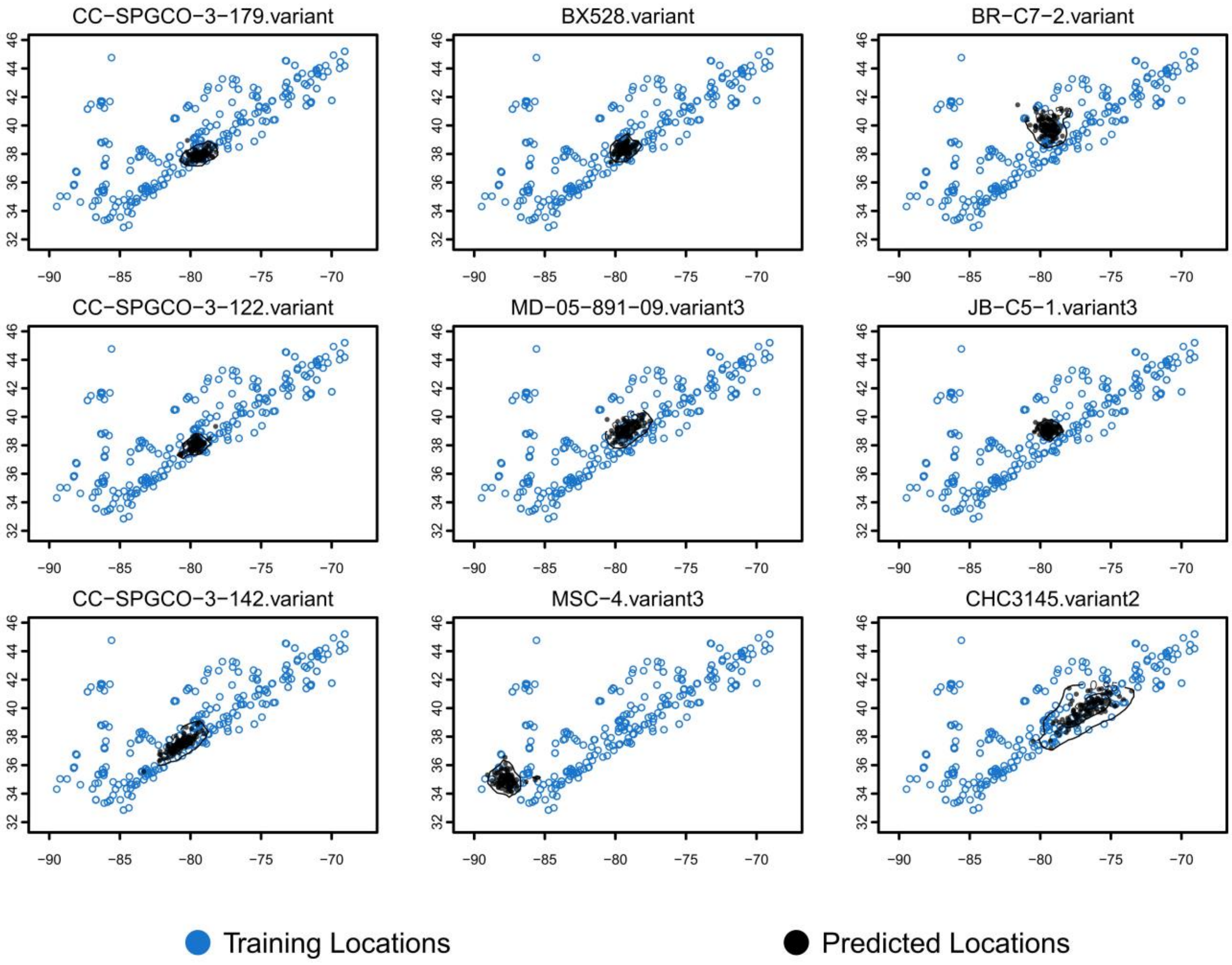

Fig. S13. Locator origin estimates for a subset of backcross and wild American chestnut trees. 100 iterations were performed and visualized here. Blue points are locations of the 356 wild American chestnut samples that were used to train Locator, and black dots are predicted locations of a single tree for each iteration. CC-SPGCO-3-179, CC-SPGCO-3122, CC-SPGCO-3-142, MSC-4, and JB-C5-1 are wild American chestnut trees, while the remaining samples are backcross trees.

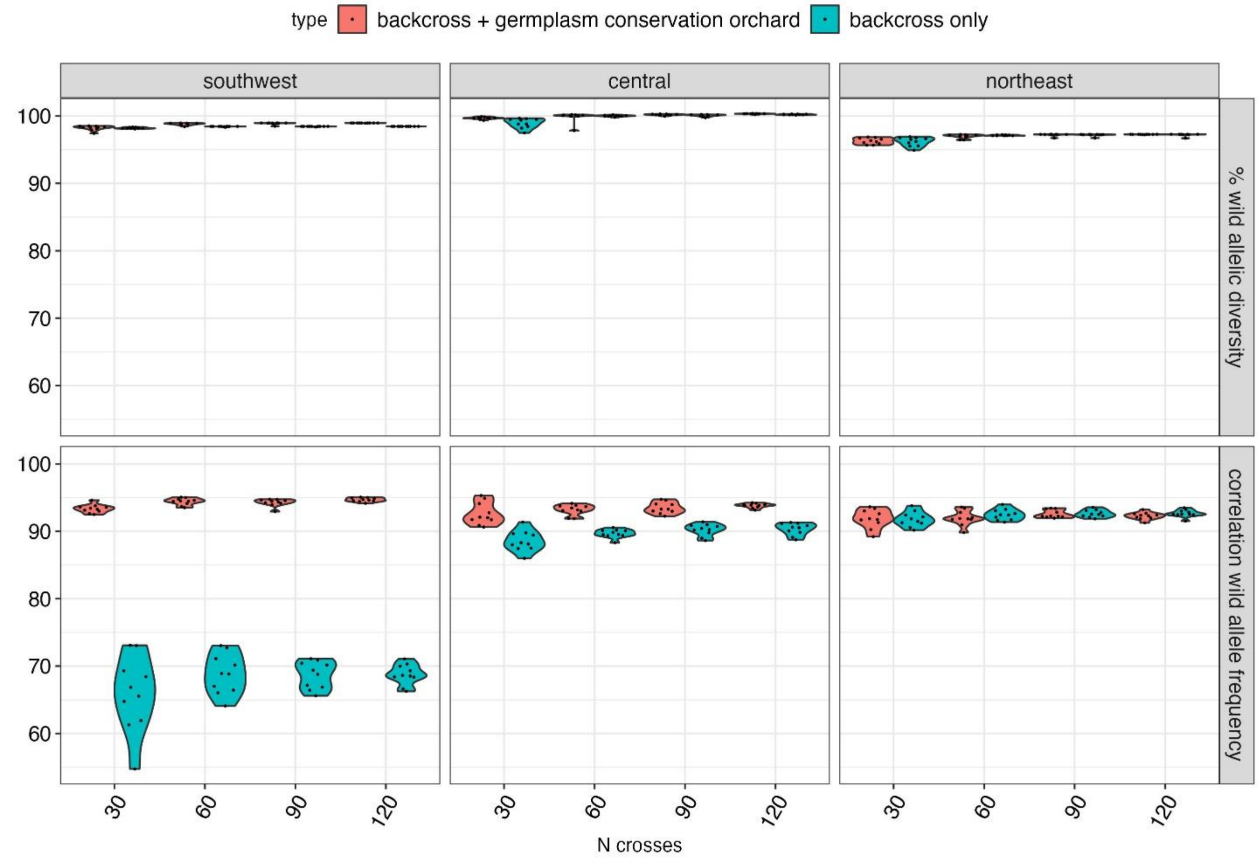

Fig. S14. Simulations of how breeding backcross trees with additional wild-type trees in TACF's germplasm conservation orchards (GCOs) influences percentage of alleles represented (top panels) and correlation with allele frequencies in the wild population (bottom panels) for 18,483 climate associated loci. For the control scenario where only backcross were intercrossed, intercrosses were simulated among 29 backcross trees from the southwest, 165 trees from the central seed zone, and 62 trees from the northeast. For the backcross + GCO scenario, intercrosses were simulated among 52 backcross + wild type trees from the southwest, 208 trees from the central seed zone, and 62 trees from the northeast. Simulations were repeated ten times for each seed zone with 30, 60, 90, or 120 random intercrosses to produce 10 progeny per cross.

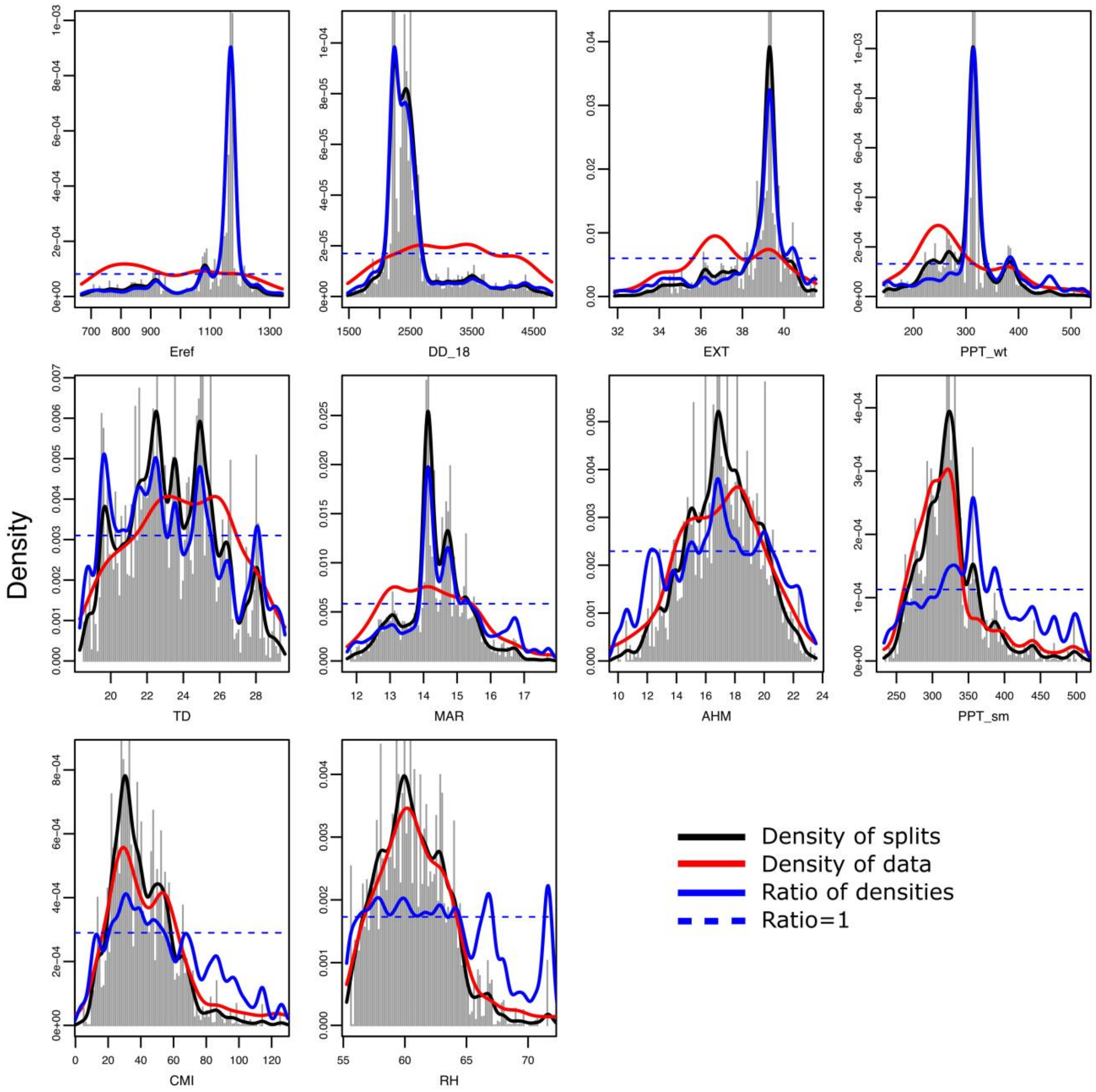

Fig. S15. Gradient Forest adaptive allelic turnover for each of the ten climate variables and 18,483 putatively adaptive loci. Spikes in the binned split importance reveal where changes in adaptive allele density are occurring along each climate gradient.
